# Blocking microglial reactivity via purinergic receptors prevents subacute cognitive deficits after TIA

**DOI:** 10.1038/s44321-026-00397-6

**Published:** 2026-03-20

**Authors:** Gemma Llovera, Steffanie Heindl, Daniel P Varga, Nikolett Lenart, Sebastian Kallabis, Vanessa Göb, David Stegner, Raphael Escaig, Leo Nicolai, Nicolai Franzmeier, Felix Meissner, Adam Denes, Arthur Liesz

**Affiliations:** 1 Institute for Stroke and Dementia Research (ISD), LMU University Hospital, LMU Munich, Munich, Germany; 2 Laboratory of Neuroimmunology, HUN-REN Institute of Experimental Medicine, Budapest, Hungary; 3 Department of Systems Immunology and Proteomics, Institute of Innate Immunity, University Hospital Bonn, Bonn, Germany; 4 Core Facility Translational Proteomics, Institute of innate Immunity, University Hospital Bonn, Bonn, Germany; 5 Julius-Maximilians University of Würzburg, Rudolf Virchow Center for Integrative and Translational Bioimaging and University Hospital Würzburg, Würzburg, Germany; 6 Medizinische Klinik und Poliklinik I, LMU University Hospital, Munich, Germany; 7 DZHK (German Centre for Cardiovascular Research), Partner Site Munich Heart Alliance, Munich, Germany; 8 Munich Cluster for Systems Neurology (SyNergy), Munich, Germany; 9 University of Gothenburg, The Sahlgrenska Academy, Institute of Neuroscience and Physiology, Department of Psychiatry and Neurochemistry, Mölndal and Gothenburg, Gothenburg, Sweden; 10 Mercator Fellow, Institute for Stroke and Dementia Research, University Hospital, LMU Munich, Munich, Germany

**Keywords:** ATP, Microglia, TIA, Purinergic Receptor, Synapse, Neuroscience

## Abstract

Our research presents a new animal model of transient ischemic attack (TIA) that mimics brief episodes without cell loss, but results in neuronal and behavioral deficits. We identified excessive microglial reactivity, driven by acute ATP release, as a key factor in post-TIA neurological deficits, which were ameliorated by inhibiting the P2Y12 receptor, a microglia-specific purinergic receptor in the brain parenchyma responsible for activity-dependent microglial cell-cell interactions. This finding suggests that modulation of microglial reactivity offers a promising strategy to prevent cognitive impairment in TIA patients, opening avenues for future research in this underexplored area.

## Synopsis

Transient ischemic attacks cause lasting cognitive deficits despite absent brain infarction. This study identifies ATP-driven microglial hyperreactivity as a key mechanism and shows that targeting microglial purinergic signaling prevents post-TIA synaptic and cognitive dysfunction.

- A refined mouse model of TIA was established that induces prolonged network dysfunction and synapse loss without neuronal death or blood–brain barrier disruption.
- Excessive microglial reactivity after TIA was driven by rapid and sustained increases in extracellular ATP.
- Microglia–neuron interactions and synaptic pruning were markedly increased via P2Y12-dependent purinergic signaling.
- Pharmacological P2Y12 inhibition normalized microglial behavior, preserved glutamatergic synapses, and prevented cognitive deficits after TIA.

## Abstract (web summary)

Transient ischemic attacks cause lasting cognitive deficits despite absent brain infarction. This study identifies ATP-driven microglial hyperreactivity as a key mechanism and shows that targeting microglial purinergic signaling prevents post-TIA synaptic and cognitive dysfunction.

## Graphical abstract

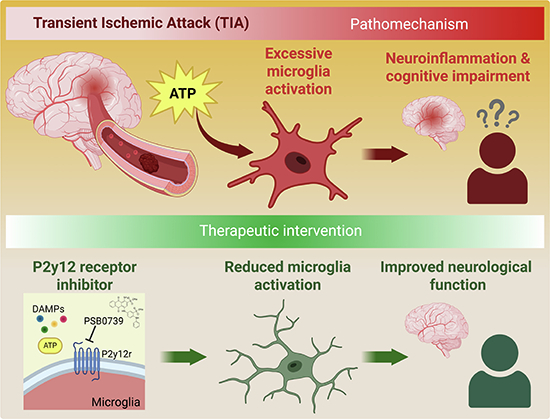

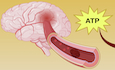

## Introduction

Transient ischemic attacks (TIAs) are a prevalent form of cerebrovascular disease in which rapid reperfusion of an occluded cerebral artery instantly restores blood flow to the affected region, resulting in a transient neurological deficit without detectable parenchymal injury (Easton et al, 2009). As such, TIA differs mainly from ischemic or hemorrhagic stroke, the other most common cerebrovascular disorders, in that it does not cause detectable structural damage to brain tissue. Nevertheless, TIAs are associated with short- and long-term risks, such as cardiovascular incidents, cognitive impairment, and increased mortality (Giles and Rothwell, 2007; Hill et al, 2004; Navis et al, 2019; Sivakumar et al, 2014; Touze et al, 2005; Wu et al, 2007). Most notably, TIA patients have a similarly increased risk of developing dementia as those who have had a stroke despite the lack of structural tissue damage after TIA (Luengo-Fernandez et al, 2013; Pendlebury et al, 2019).

Annually, around 500,000 TIA cases are reported in the United States alone and up to 60 cases per 100,000 across Western countries (Brown et al, 1998; Cancelli et al, 2011; Degan et al, 2017), marking TIA as an early warning sign critical for preventing stroke and dementia. However, current therapies for TIA are limited to control of cardiovascular risk factors (Johnston et al, 2018; Powers et al, 2019; Wang et al, 2013), while pathomechanisms of TIA and their role in post-TIA cognitive deficits remain elusive and so far unexplored.

## Results and discussion

**Figure 1:**
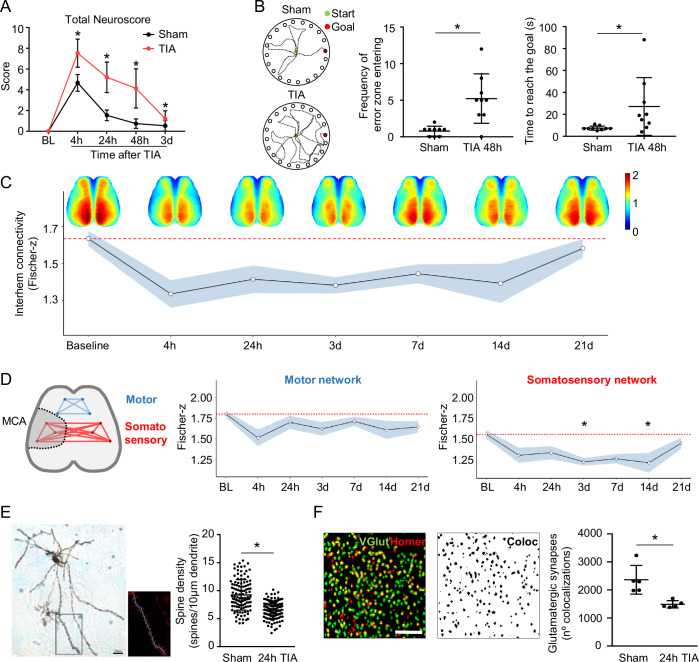
Brief transient ischemia, in the absence of tissue injury, leads to prolonged neuronal dysfunction. (**A**) Neuroscore at different time points after TIA in male animals (*n* = 15 per group; 4, 24, and 48 h:*p* value <0.0001, 3 d:*p* value = 0.023) and (**B**) mean average of mice trajectories to reach the goal; frequency of errors (*p* value = 0.0023) and time to reach the goal (*p* value = 0.0070) in the Barnes Maze test at 48 h after TIA in males (*n* = 9 per group). (**C**) Global interhemispheric connectivity and (**D**) network connectivity for motor and somatosensory networks at indicated time points before in males (Baseline and red dotted line) and after TIA (*n* = 8 per time point; 3 d:*p* value = 0.032 and 14 d:*p* value = 0.044) (MCA middle cerebral artery vascular territory). (**E**) Representative image of Golgi-Cox stained pyramidal neuron 24 h after TIA in males, scale bar = 10 µm. Magnification show an example of 3D reconstruction for a dendrite section with spines used for their quantification (*n* = 5 per group; *p* value <0.0001). (**F**) Representative particle image of presynaptic terminals by VGlut1 (green) and postsynaptic terminals by Homer1 (red), as used for quantification of colocalized presynaptic and postsynaptic particles (black) 24 h after TIA in males (*n* = 5 per group; *p* value = 0.0079), scale bar = 10 µm. In (**C**, **D**): black line: mean and gray: Standard Error. Statistical tests: (**A**) two-way ANOVA, corrected for multiple comparisons using two-stage step-up method of Benjamin Kriegel. (**B**, **E**, **F**) *T*-test. (**C**) repeated measure of ANOVA followed by Turkey’s post hoc test. Error bars indicate ±SD. *p < 0.05. Source data are available online for this figure.

**Figure EV1:**
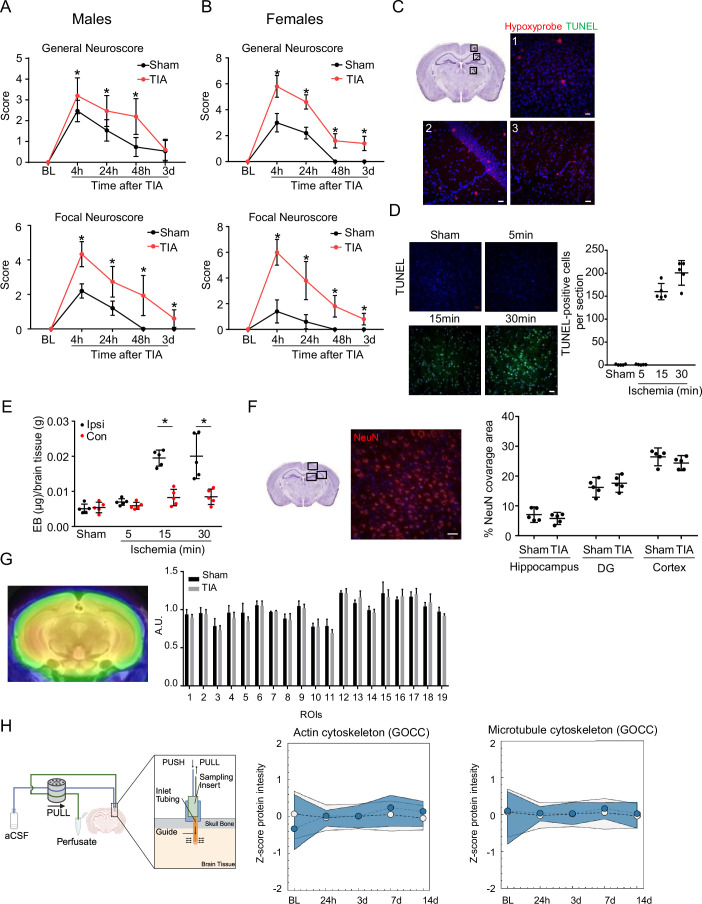
Characterization of the TIA model. General and focal Neuroscore at different time points after TIA in (**A**) males (*n* = 15 per group; General Neuroscore: 4 h: *p* value = 0.0004, 24 and 48 h:*p* value <0.0001; Focal Neuroscore: 4, 24, and 48 h:*p* value <0.0001, 3 d:*p* value = 0.0012) and (**B**) females (*n* = 5 per group; General Neuroscore: 4, 24, 48 h and 3 d:*p* value <0.0001; Focal Neuroscore: 4, 24 h: *p* value <0.0001, 48 h:*p* value = 0.0002, 3 d:*p* value = 0.045). (**C**) Representative image for brain hypoxia (hypoxyprobe in red) and cell death (TUNEL^+^ in green) 24 h after TIA in males in cortex (1), hippocampus (2) and striatum (3), scale bar = 20 µm. (**D**) Representative TUNEL^+^ staining and quantification 24 h after different MCA occlusion times in males (*n* = 5 per group), scale bar = 20 µm. (**E**) Blood–brain barrier integrity was measured by extravasated Evans Blue per gram of the brain tissue 24 h after different ischemia times in males. Black = ipsilateral hemisphere, red = contralateral hemisphere (*n* = 5 per group; 15 and 30 min ischemia: *p* value = 0.0079). (**F**) Representative image and analysis of neural cells (NeuN+) in different brain regions in males (DG dentate gyrus), scale bar = 20 µm. (**G**) Representative image and analysis of glucose metabolism using in vivo positron-emission tomography 24 h after TIA or Sham males (*n* = 6 per group). 1-Right striatum, 2-Left striatum, 3-Cortex, 4-Right hippocampus, 5-Left hippocampus, 6-Thalamus, 7-Carebelum, 8-Basal forebrain/septum, 9-Hypothalamus, 10-Right amygdala, 11-Left amygdala, 12-Brainstem, 13- Central gray, 14-Superior colliculi, 15-Olfactory bulb, 16-Midbrain right, 17-Midbrain left, 18- Inferior colliculi left, 19-Inferior colliculi right. (**H**) Representative scheme of the microperfusion system and protein intensity over time before (BL) and after TIA in males (*n* = 5 per group). Statistical tests: (**A**, **B**, **E**, **F**) two-way ANOVA, corrected for multiple comparisons using two-stage step-up method of Benjamin Kriegel. (**H**) Two-way ANOVA, Fisher’s exact testing. Error bars indicate ±SD. *p < 0.05. Source data are available online for this figure.

**Figure EV2:**
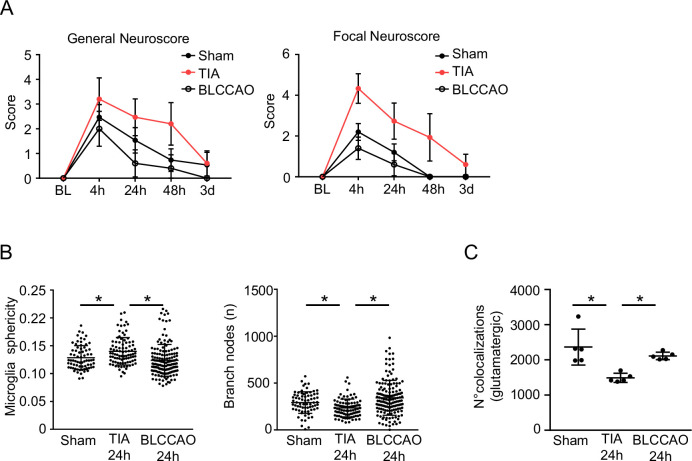
TIA vs BLCCAO. (**A**) General and focal Neuroscore at different time points after TIA, Sham and BLCCAO male animals (*n* = 5 per group). (**B**) Microglia morphology analysis (sphericity and branch nodes) at different time points 24 h after TIA, Sham and BLCCAO male animals (*n* = 5 per group; Microglia sphericity: TIA:*p* value = 0.0123 and BLCCAO:*p* value <0.0001), Branch nodes: TIA:*p* value = 0.0022 and BLCCAO:*p* value <0.0001. (**C**) Quantification of colocalized glutamatergic presynaptic and postsynaptic particles 24 h after TIA, Sham, and BLCCAO male animals (*n* = 5 per group; TIA:*p* value = 0.0140 and BLCCAO:*p* value = 0.0400). Statistical tests: (**A**–**C**) two-way ANOVA, corrected for multiple comparisons using two-stage step-up method of Benjamin Kriegel. Error bars indicate ±SD. *p < 0.05.

In the past decade, the clinical diagnosis of TIA has faced a paradigm shift from “time-based” (focal deficits for less than 1 h) to a “tissue-based” definition (acute neurological symptoms without brain infarction) (Degan et al, 2017; Hurford et al, 2019). Some previous work have described different mouse model of TIA (Pedrono et al, 2010; Quenault et al, 2017; Wang et al, 2020), but its characterization, in our view, was not sufficient. Consequently, given our experience with murine stroke models, we took the classic model of intraluminal middle cerebral artery (MCA) occlusion (Llovera et al, 2021) as a reference, and adapted it to establish a model of TIA and characterized it in more detail. A brief five-minute occlusion of the MCA was sufficient to induce significant behavioral deficits both displaying general and focal deficits both in male and females (Figs. 1A and EV1A,B) as well as impaired spatial memory up to two days after TIA in males (Fig. 1B). However, these brief ischemic episodes leading to transient tissue hypoxia (Fig. EV1C) did not induce any detectable cell death, change in blood–brain barrier permeability, neuronal loss, changes in glucose metabolism by in vivo positron-emission tomography or up-regulation of cytoskeleton proteins in the extracellular space, confirming the absence of any structural tissue damage (Fig. EV1D–H). We also performed cerebral hypoperfusion for 5 min using a bilateral common carotid artery occlusion (BLCCAO) model, which was not sufficient to induce behavioral changes, microglia activation or a decrease in the number of synapses (Fig. EV2A–C). Therefore, this model closely resembles the pathophysiological definition of TIA (Perry et al, 2022) and allows in-depth functional studies.

After observing neurological deficits, we set out to explore the pathophysiological mechanisms mediating prolonged functional deficits after TIA by performing repetitive wide-field neuronal calcium imaging to analyze cortical network function (Cramer et al, 2019). To our surprise, we found that a transient ischemic stroke led to a significant reduction in global interhemispheric network connectivity for more than 14 days, driven by a specific lack of connectivity in the network of the somatosensory cortex supplied by the MCA, whereas other cortical areas not supplied by the affected MCA did not suffer significant alterations (Fig. 1C,D). These results suggest a very prolonged and specific effect of the brief TIA period on cortical network function in the affected vascular brain area (somatosensory cortex). As we found stable neuronal survival (Fig. EV1F), we analyzed subcellular remodeling using spine density by histology, noting a significant reduction in dendritic spine density 24 h after TIA (Fig. 1E). Correspondingly, glutamatergic synapse density in the somatosensory cortical area of the MCA territory was significantly reduced (Fig. 1F).

**Figure EV3:**
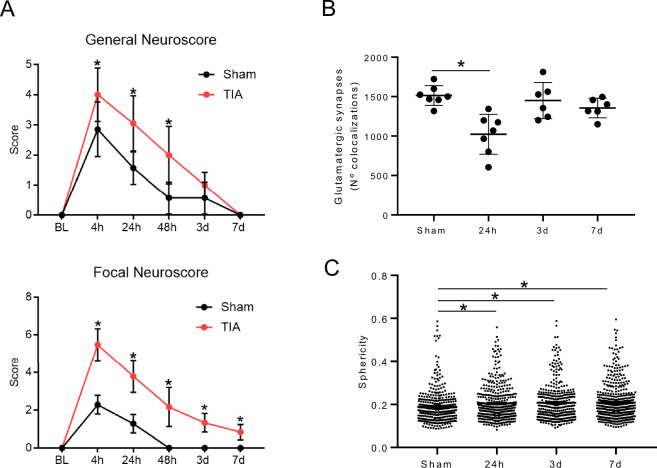
TIA in aging. (**A**) General and focal Neuroscore at different time points after TIA in male aging animals (20 months) (Sham: *n* = 7; TIA: BL-24 h *n* = 19, 48 h-3d *n* = 12, 7 d *n* = 6; General neuroscore: 4 h: *p* value = 0.0002, 24 and 48 h:*p* value <0.0001; Focal Neuroscore: 4, 24, and 48 h:*p* value <0.0001, 3 d:*p* value = 0.0034). (**B**) Quantification of colocalized glutamatergic presynaptic and postsynaptic particles at different time points after TIA in male aging animals (20 months) (Sham: *n* = 7; TIA: 24 h *n* = 7, 3d–7d *n* = 6; 24 h:*p* value = 0.0003). (**C**) Microglia morphology analysis (sphericity) at different time points after TIA in male aging animals (20 months) (Sham: *n* = 7; TIA: 24 h *n* = 7, 3d–7d *n* = 6; 24 h:*p* value = 0.0083, 3 d:*p* value = 0.0027, 7 d:*p* value = 0.0011). Statistical tests: (**A**–**C**) two-way ANOVA, corrected for multiple comparisons using two-stage step-up method of Benjamin Kriegel. Error bars indicate ±SD. *p < 0.05.

Age is a crucial factor in transient ischemic attacks (TIAs) because it significantly increases both the risk of having a TIA and the likelihood of developing other health problems that contribute to vascular events (Khare, 2016). As people age, they often accumulate diseases such as hypertension, diabetes, and atrial fibrillation, which increase the risk of TIA (Ippen et al, 2021). Consequently, older people who suffer a TIA tend to have worse outcomes, such as higher rates of subsequent stroke, rehospitalization, and shorter life expectancy (Fasth et al, 2021; Gattellari et al, 2012). Recognizing age as a key comorbidity is vital for effective risk assessment and personalized management of TIA patients. For this reason, we decided to test the effects of TIA in older animals to see if we could observe an exacerbated effect of the TIA phenotype. We observed a similar but more pronounced effect in older animals (20-month-old), both in their Neuroscores and in the reduction of synapses; however, this effect remained transient (Fig. EV3A–C). Although cohorts cannot be directly compared, we observed a reduction in glutamatergic synapses in sham-aged animals compared to sham-young animals (compare Figs. EV3B and 1F), indicating that aging alone already has an effect on synaptic density.

**Figure 2:**
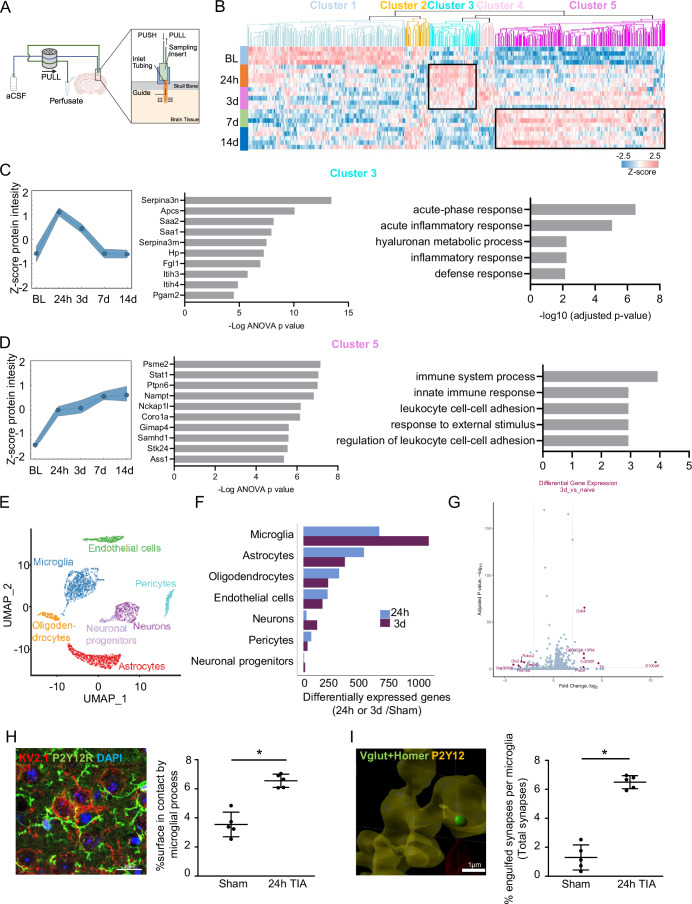
TIA triggers a cortical proinflammatory environment and microglial reactivity. (**A**) Representative scheme of the microperfusion system. (**B**) Heat map of all ANOVA+ proteins of the LC-MS analysis of the perfusate products, (**C**) protein intensity over time, top ten upregulated genes and pathway analysis from cluster 3 and (**D**) cluster 5 in males (*n* = 5 per group). (**E**) Uniform manifold approximation and projection (UMAP) plot, colored by identified clusters (different cell types). (**F**) Number of differentially expressed genes (*p* < 0.05) at 24 h and 3 d after TIA in each cell type compared to control (naïve) in males (*n* = 3 per group). (**G**) Volcano plot of differentially regulated genes in microglia 3 d compared to naïve animals. Cut-off for illustrating gene names: Log2FC >|2| illustrated by vertical gray dashed lines, adjusted *p* value: >−log10(0.05) illustrated by horizontal gray dashed line (*n* = 3 per group). (**H**) Representative image and quantification of microglia (P2Y12R^+^) and neuron soma (KV2.1^+^) contacts 24 h after TIA in males (*n* = 5 per group; *p* value = 0.0079), scale bar = 20 µm. (**I**) Representative image of microglia (P2Y12^+^) and synapse (Vglut^+^Homer^+^) staining for synapse engulfed quantification 24 h after TIA in males (*n* = 5 per group; *p* value = 0.0079), scale bar = 1 µm. Statistical tests: (**B**–**D**) Two-way ANOVA, Fisher’s exact testing. (**G**) Wilcoxon rank sum test + Bonferroni correction. (**H**, **I**) Two-way Student’s *t*-test. Error bars indicate ±SD. *p < 0.05. Source data are available online for this figure.

Since the underlying cause of the observed TIA phenotype was unclear, we set out to look for changes in the extracellular compartment to better understand the possible mechanisms involved. Using microfluidic perfusion of the extracellular space (Birngruber et al, 2013), in the somatosensory cortex, we sampled up to 14 d after TIA. These perfusates were used for proteomic analyses by liquid chromatography coupled with mass spectrometry (LC-MS). We identified a specific protein cluster regulated in the acute to subacute phase (cluster 3) and a group of secreted proteins upregulated in the chronic phase (cluster 5) (Fig. 2A,B). Closer examination of groups 3 and 5 revealed a peak of protein intensity at 24 h in group 3, while group 5 showed a steady increase in protein intensity expression over time (Fig. 2C,D), reinforcing our previous observation (Fig. 2A,B). Further analysis of the pathways of the ten most upregulated genes in each of these two groups indicated a large number of genes related to stress and inflammatory response, indicating a chronic inflammatory response after TIA (Fig. 2C,D). The key limitation is that the proteomics analysis of the microfluidic perfusate was biased towards highly abundant proteins (such as albumin and hemoglobin), which dominate the sample’s protein content (the top 20 proteins constitute almost 50% of the total abundance). This high dynamic range in protein concentration, combined with the limited sample volume, significantly reduced the sensitivity for detecting low-abundant, yet biologically critical, proteins like cytokines, potentially leading to an incomplete or biased picture of the extracellular protein environment. Future studies would benefit from using techniques, such as the Seer Proteograph technology, to circumvent this high dynamic range challenge.

**Figure EV4:**
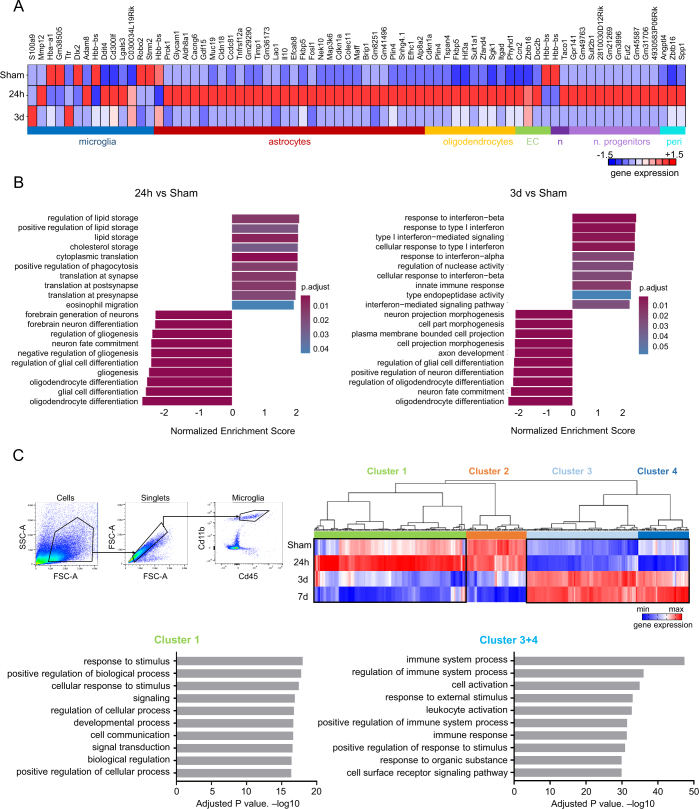
The impact of TIA on microglia transcriptome. (**A**) Heat map of normalized and scaled (*z*-score) gene expression of significantly differentially up- and down-regulated genes in each cell type at 24 h and 3 d after TIA compared to Sham in males (*n* = 3 per group). (**B**) Gene set enrichment analyses (Biological Processes) of differentially regulated genes in microglial cells at 24 h and 3 d compared to Sham in males (*n* = 3 per group). (**C**) Representative FACS plot sort strategy for microglia-like cells isolation, cluster heat map showing ANOVA+ genes from Nanostring analysis from isolated microglia-like cells at different time points after TIA and pathway analysis from selected cluster 1 (*n* = 3 per group) and cluster 3 + 4 (*n* = 3 per group) in males. Statistical tests: (**A**) Wilcoxon rank sum test + bonferroni correction. (**B**) Genes ranked by log2 fold change, permutation-based GSEA, Benjamini–Hochberg correction, (**C**) Data were analyzed by ROSALIND® (https://rosalind.bio/). Significantly regulated genes were identified by ANOVA multiple sample testing (S0 = 0.1, permutation-based FDR = 0.05)

To identify which cell populations in the cortex were most transcriptomically affected after TIA, we performed single-cell RNA sequencing of cells isolated from cortical tissue homogenates in control animals and at 24 h and 3 d after TIA. Surprisingly, neurons showed only a very minor transcriptional regulation, while the largest number of differentially regulated genes was detected in the microglia population, particularly at 3 d after TIA (Fig. 2E,F). Further analysis of genes differentially regulated in the microglial cell population revealed S100a, Ddit4 and Cd300lf being among the most highly upregulated genes at 3 d after TIA. These genes are pivotal in responding to cellular stress, including hypoxia and neuroinflammation (Denstaedt et al, 2018; Perez-Sisques et al, 2021; Voss et al, 2015) (Fig. 2G), which was also reflected by the corresponding pathway analysis of regulated genes in the microglia cell cluster (Fig. EV4A,B).

**Figure EV5:**
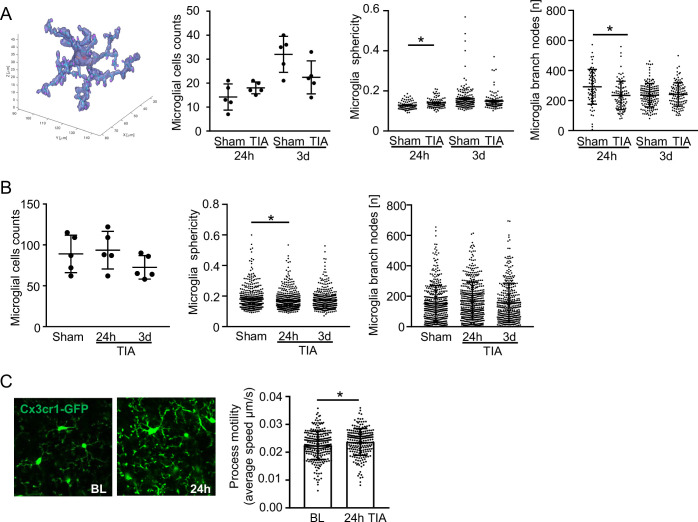
The impact of TIA on microglia morphology. (**A**) Representative image of 3D reconstructed microglia for microglia cells counts and microglia morphology analysis for two representative features: sphericity and branches nodes at 24 h after TIA (*n* = 5 per group; sphericity 24 h:*p* value = 0.019; branch nodes 24 h:*p* value = 0.022) in males and (**B**) females (*n* = 5 per group; sphericity 24 h:*p* value = 0.0032). (**C**) Representative images of in vivo two-photon imaging of the microglia process motility and quantification at baseline (BL) and 24 h after TIA in males (*n* = 8 per group; 24 h:*p* value = 0.025). Statistical tests: (**A**, **C**) two-way ANOVA, corrected for multiple comparisons using two-stage step-up method of Benjamin Kriegel. (**B**) two-way Student’s *t*-test. Error bars are mean ± SD. *P < 0.05. Source data are available online for this figure.

Given that microglial cells showed the most significant transcriptional changes, we focused on studying these cells in more detail. To do so, we performed a specific analysis of microglia using Nanostring technology, examining selected microglial populations up to 7 days after TIA, which confirmed long-lasting transcriptional changes with proinflammatory polarization in microglial reactivity still at 7 d post-TIA (Fig. EV4C). Based on these transcriptomic findings indicating an important role of microglia in the post-TIA brain, we further investigated the cellular function and morphology of microglia. Structural analysis of microglial cells in the somatosensory cortex, by automated 3D-morphometry (Heindl et al, 2018), identified a significant change in microglial morphology only 24 h after TIA, which suggests a transient reactive phenotype in males (Fig. EV5A) and also in females (Fig. EV5B). Correspondingly, in vivo analysis of microglia confirmed a significant increase in process motility (Fig. EV5C)—a microglial phenotype that has been previously associated with altered cell–cell interaction and potential neurotoxicity (Block et al, 2007; Cserep et al, 2020). Activation of microglia after a TIA can also be observed in older animals (20 months old), but here the microglial cells remained activated for up to 7 days after the TIA (Fig. EV3C).

Since we detected neuronal dysfunction (Fig. 1) as well as microglial reactivity in both histology and transcriptome (Figs. EV4 and 5), we decided to investigate the two main functions of microglial cells already described in the literature, neuronal surveillance (Cserep et al, 2020) and synapse pruning (Hong et al, 2016). Therefore, we further investigated the microglia–neuron interaction as a potential basis of prolonged neuronal dysfunction after TIA. Specifically, microglial interaction with neuronal somata (termed somatic purinergic junctions) were identified to be critical for microglial sensing of neuronal health (Cserep et al, 2022); this interaction between P2Y12^+^ microglia and Kv2.1^+^ neurons was substantially increased after TIA (Fig. 2H). Similarly, the contact between microglia and Vglut^+^Homer1^+^ excitatory synapses was also substantially increased after TIA (Fig. 2I), suggesting increased microglia–neuron interaction as a potential contributor to prolonged neuronal dysfunction after TIA.

**Figure 3:**
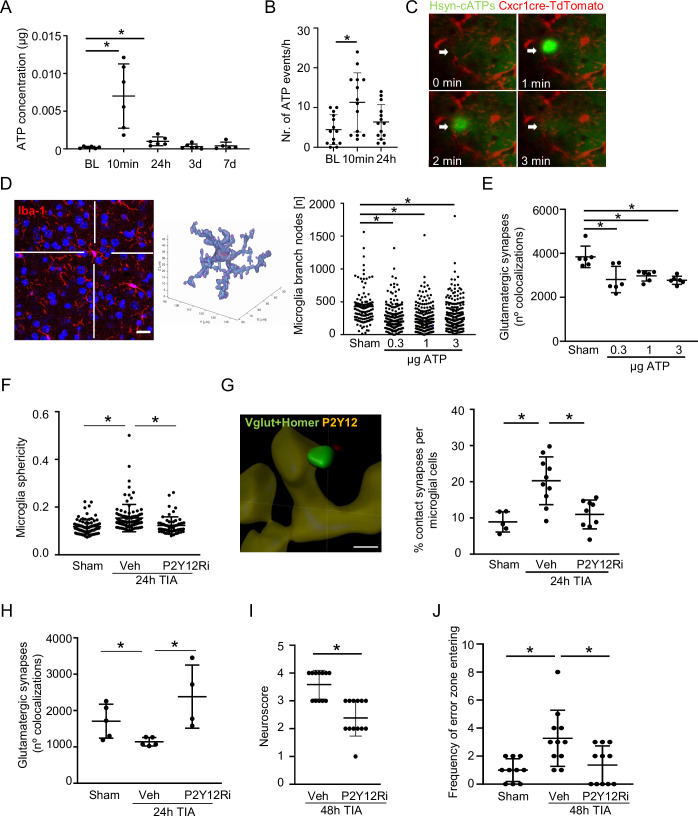
P2Y12R inhibition confirms ATP-mediated microglia activation after TIA. (**A**) Luciferace assay to detect ATP concentration from peri-lesional perfusate (see Fig. 2A) at before (BL) and at indicated time points after TIA in males (*n* = 6 per group; 10 min:*p* value = 0.0002, 24 h: *p* value = 0.0231). (**B**) Quantification of extracellular ATP (hsyn-cATPs) using in vivo two-photon imaging before (BL) and at indicated time points after TIA (*n* = 18 per group; 10 min:*p* value <0.01) and (**C**) microglial processes (Cx3Cr1cre-TdTomato) recruitment to focal increase in extracellular ATP within 3 min after TIA in males. (**D**) Representative image of Iba1+ microglia cell and 3D reconstructed microglia for branches nodes analysis 24 h after cisterna magna injection of different ATP concentrations in males (*n* = 6 per group; 0.3, 1, and 3 µg ATP:*p* value <0.0001), scale bar = 20 µm. (**E**) Quantification of colocalized presynaptic and postsynaptic particles 24 h after cisterna magna injection of different ATP concentrations in males (*n* = 6 per group; 0.3, 1, and 3 µg ATP:*p* value <0.0001). (**F**) Microglia sphericity (*n* = 5 per group; Veh:*p* value <0.0001 and P2Y12Ri:*p* value <0.0001), (**G**) synapse (Vglut^+^Homer^+^) contacts with microglial cells (P2Y12^+^) (*n* = 10 per group; Veh:*p* value <0.0033 and P2Y12Ri:*p* value <0.0035) (scale bar = 2 µm) and (**H**) glutamatergic synapse counts (*n* = 5 per group) in sham and 24 h after TIA in vehicle (Veh) or P2Y12Ri (-inhibitor) treated animals (in males’ mice); Veh:*p* value = 0.0140 and P2Y12Ri:*p* value = 0.0155. (**I**) Neuroscore at 48 h after TIA in vehicle (Veh) or P2Y12Ri (-inhibitor) treated animals (*n* = 10 per group; *p* value = 0.0002) and (**J**) Barnes Maze test (*n* = 10 per group) in Sham and 48 h after TIA in vehicle (Veh) or P2Y12Ri (-inhibitor) treated animals (in males’ mice); Veh:*p* value = 0.0083 and P2Y12Ri:*p* value = 0.0469. Statistical tests: (**A**–**J**) two-way ANOVA, corrected for multiple comparisons using two-stage step-up method of Benjamin Kriegel. (I) Mann–Whitney *U*-test. Error bars indicate ±SD. *p < 0.05. Source data are available online for this figure.

Next, we aimed to identify the molecular mechanisms leading to the excessive microglial reactivity to the brief ischemic period during TIA. The rapid depletion of oxygen and glucose during a TIA leads to a significant increase in extracellular ATP, which acts as a crucial damage-associated molecular pattern (DAMP). When we checked for extracellular ATP concentration after TIA, we observed a rapid and massive increase within minutes after TIA which remained elevated until 24 h post-TIA (Fig. 3A). This phenomenon was confirmed by in vivo imaging using an extracellular ATP reporter system by AAV-mediated expression of the hsyn-cATPs sensor in the MCA-supplied area of Cx3cr1-tdT microglial reporter mice, which revealed an increase of focal ATP bursts after TIA (Fig. 3B). This surge in extracellular ATP was associated with rapid recruitment of microglial processes within 60-180 s after the ATP burst (Fig. 3C; Movie EV1). Remarkably, these events influence microglial function through P2Y12R and CX3CR1 pathways (Haynes et al, 2006; Huang et al, 2024). Additionally, these pathways contribute to the generation of focal ATP events, which could be highly relevant for transient ischemic attacks (TIA) and other forms of relatively subtle injury. Recent data show that microglial process recruitment to focal ATP events below 1 µM takes place via P2Y12R-mediated effects that have major effects on microglial activity and cell morphology (Berki et al, 2024). To test the causal role of increased extracellular ATP concentration for microglial reactivity and neuronal dysfunction, we injected ATP into the cisterna magna (CM) of naïve mice, which was sufficient to dose-dependently induce a similar phenotype on microglial morphology and glutamatergic synapse loss as TIA (Fig. 3D,E).

**Figure EV6:**
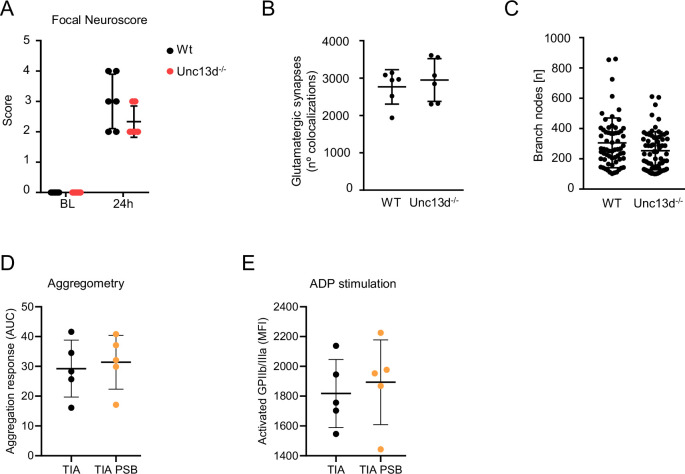
Platelets are not critical after a TIA. (**A**) Focal neuroscore, (**B**) glutamatergic synapses, and (**C**) microglia branch nodes analysis of Unc13KO and littermates wild-type (WT) animals 24 h after TIA in males. (**D**) Platelet aggregation and (**E**) activation in TIA in vehicle (Veh) or P2Y12Ri (-inhibitor) treated male animals (*n* = 5 per group). Statistical tests: (**A**–**E**) two-way Student’s *t*-test. Error bars are mean ± SD.

This sudden increase in ATP can originate from multiple sources: acutely stressed or damaged neurons, which release ATP into the synaptic cleft, and astrocytes, which respond with a rapid release of gliotransmitters. Most critically, this high concentration of extracellular ATP rapidly activates resident microglia through purinergic receptors, initiating a robust innate immune and inflammatory response (Rodrigues et al, 2015). While the exact cellular source of the extracellular ATP increase remains unclear, we postulate that astrocytes, neurons, and tentatively other resident brain cells contribute to its surge after TIA. In the injured brain, the main source of focal ATP events are considered to be astrocytes (Chen et al, 2024). This assumption is supported by the observation that the deficiency in ATP-release from platelets—another potential major source of rapid ATP release from the intravascular compartment, particularly in vascular injury—does not affect the post-TIA phenotype in Unc13d-deficient mice—mice with Munc13-4-deficient platelets, which are not able to degranulate the dense granules, so there is no ATP release (Fig. EV6A–C).

ATP sensing by purinergic receptors is a well-described mechanism of microglial activation with P2Y12 as one of the most abundant and microglia-specific purinergic receptors (Cserep et al, 2020; Davalos et al, 2005; Horvath et al, 2014). Therefore, we tested the potential of a pharmacological P2Y12 receptor blockade (P2Y12Ri), PSB-0739, on preventing post-TIA microglial effects. Indeed, P2Y12Ri treatment prevented morphological changes of microglia and normalized microglia-synapse interaction frequency (Fig. 3F,G), which was associated with significantly increased numbers of glutamatergic synapses after TIA in the treatment group (Fig. 3H). Importantly, inhibition of purinergic signaling after TIA was able to significantly improve focal neurological deficits and prevent the cognitive deficits in spatial memory after TIA (Fig. 3I,J). In addition, we found no effect on platelet aggregation in the systemic circulation (Fig. EV6D,E).

This robust study established a well-characterized mouse model of transient ischemic attack (TIA) that successfully replicates the tissue-based clinical definition, demonstrating prolonged functional deficits (synapse loss and network disconnectivity) without structural damage. The core strength lies in identifying a novel pathogenic mechanism after TIA: the TIA induces a rapid increase in extracellular ATP, triggering P2Y12 receptor-mediated microglial activation and subsequent synaptic pruning, a pathology which was reversed by P2Y12 receptor inhibition. However, the precise cellular source of the critical extracellular ATP surge remains unclear. Additionally, the study is limited by the absence of an a priori power calculation; our sample size was not prospectively determined to detect a specific effect size. This approach may have implications for the statistical power of the study and suggests that the results, particularly non-significant findings, should be viewed as exploratory. The study’s main contribution lies in generating new hypotheses, which can now be tested in future, adequately powered studies. This will ensure that our findings are validated and their broader significance is firmly established.

Collectively, our findings support targeting purinergic microglial reactivity as a promising immunomodulatory approach to prevent secondary neurological deterioration in patients with transient ischemic attacks. We anticipate that our findings, along with our extensively characterized animal model of TIA, will lay the groundwork for future investigations into the mechanisms and therapeutic interventions for this highly prevalent yet underexplored neurological condition.

## Methods

**Table:** Reagents and tools table

Reagent/resource	Reference or source	Identifier or catalog number
**Experimental models**		
C57BL6/J (*M.Musculus*)	Charles river	Strain: 632
C57BL/6J-Tg (Thy1-GCaMP6s) GP4.12Dkim/J (*M.Musculus*)	Dana et al, 2014	Institute for Stroke and Dementia Research, Munich
CX3CR1^GFP/+^ (*M.Musculus*)	Jaxson	Strain: 005582
CX3CR1^tdTomato^ (*M.Musculus*)	Jaxson	Prof.A. Denes at the HUN-REN Institute of Experimental Medicine in Budapest
Munc13-4-null (Unc13dKO) (*M.Musculus*)	Stegner et al, 2013	Prof. D. Stegner at the Institute for Experimental Biomedicine, University of Würzburg
**Recombinant DNA**		
**Antibodies**		
Guinea pig anti-VGlut1	Millipore	AB5905
Chicken anti-Homer	Synaptic Systems	160006
rabbit anti-P2Y12	AnaspecInc	AS-55043A
mouse anti-KV2.1	NeuroMab	75-014
rabbit anti-Iba1	Wako	019-19741
anti-NeuN antibody	MerkMillipore	MAB377
AF488 goat anti-Guinea pig	Molecular Probes	A-11073
AF647 goat anti-Chicken	Invitrogen	A-21449
AF488 goat anti-rabbit	Thermo Fisher Scientific	A-11034
AF647 goat anti-mouse	Invitrogen	A-21235
AF594 goat anti-rabbit	Invitrogen	A-11012
AF488 goat anti-mouse	Invitrogen	A11001
DAPI (4’-6-Diamidino-2-Phenylindole-dihydrochloride)	Thermo Fisher Scientific	D3571
**Oligonucleotides and other sequence-based reagents**		
**Chemicals, enzymes and other reagents**		
P2Y12R inhibitor, PSB-0739	Bio-Techne Corp	3983
artificial cerebral spinal fluid (aCSF)	Bio-Techne Corp	3525
Adenosine 5-triphosphate disodium salt hydrate	Sigma	A1852-1VL
Ambion^TM^ Nuclease-Free water	Invitrogen	AM9937
green ATP sensor	WZ Biosciences	YL006006-AV9
[18 F]FDG tracer	LMU University Hospital	N/A
Evans blue	Sigma-Aldrich	E2129
formamide	Roth	6949.1
Paraformaldehyde	Morphisto	Cat# 11762.00100
Triton X-100	Sigma-Aldrich	Cat# 10241957
Tween 20	Roth	Cat# 9127.1
Bovine Serum Albumin	Sigma-Aldrich	Cat# 9048-46-8
cold fish skin gelatine	Sigma-Aldrich	G7041
TUNEL apoptosis detection kit	Millipore	S7110
Hypoxyprobe-1 Green Kit	HPI	HP6-XXX
Donkey serum	Abcam	Cat# ab7475
Goat serum	Thermo Fisher Scientific	Cat# 16210064
Aldehyde fixative solution	Bioenno	003780
Impregnation solution	Bioenno	003760
DMEM	Invitrogen	Cat# 12634-010
Fetal calf serum (FCS)	Gibco	Cat# 105000-064
Digestion mix [DMEM + 10%FCS + 0.4%DNASEI	Roche	11284932001
CollagenaseD	Roche	11088866001
Agarose	VWR Chemicals	9012-36-6
**Software**		
GraphPad Prism 7/9/10	GraphPad Software Inc.	N/A
FIJI (ImageJ 2, Version 2.0.0-rc-69/1.52p & 2.14.0/1.54f)	NIH	N/A
Imaris (Version 8.4.0)	Bitplane	N/A
ZEN black edition	Zeiss	N/A
MATLAB (R2016b)	Mathworks	N/A
R version 4.0.3/4.3.1	CRAN	N/A
MES	Femtonics	v.5.3560
Microglia Morphology Quantification Tool (MMQT)	Heindl & Gesierich et al	N/A
CellRanger v.7.1.0/v.8.0.0)	10x Genomics	N/A
**Other**		
Silicon-coated filament	Doccol	602112PK5Re
Silicon-coated filament	Doccol	602156PK10
Laser Doppler	Perimed	
Confocal microscope	Zeiss	Zeiss 880
Spectrophotometer plate reader at 620 nm	Bio-Rad	168-1130
Barnes Maze	Noldus	
Quick Base	Parkell C&B metabond	S398
L-Powder clear	Parkell C&B metabond	S399
Universal Catalyst	Parkell C&B metabond	S371
Artificial cerebrospinal fluid (aCSF)	https://www.alzet.com/guide-to-use/preparation-of-artificial-csf/	
Guide/Dummy	Joanneum research	cOFM-GD-2-1
Sampling insert	Joanneum research	cOFM-S-3
Perfusate bag 10 ml	Joanneum research	OFM-BAG
Low-binding tubing	Joanneum research	OFM-PP2-100-LB
Microperfusion pump	Joanneum research	MPP102-II-PC

### Mice

The experiments were conducted following national guidelines for the use of experimental animals, and all protocols were approved by the German governmental committees (Regierung von Oberbayern, Munich, Germany and Regierung von Unterfranken, Würzburg, Germany). Wild-type C57BL6/J mice were purchased from Charles River, C57BL/6J-Tg (Thy1-GCaMP6s) GP4.12Dkim/J (Dana et al, 2014) heterozygous mice were bred at the Institute for Stroke and Dementia Research, Munich. CX3CR1^GFP/+^(JAX stock no: 005582), CX3CR1^tdTomato^ (offsprings of JAX stock no: 020940 with JAX stock no: 007905) were bred by the laboratory of Prof. A. Denes at the HUN-REN Institute of Experimental Medicine in Budapest. Munc13-4-null (Unc13dKO) mice (Stegner et al, 2013) and WT-littermate controls were bred by Prof. D. Stegner at the Institute for Experimental Biomedicine, University of Würzburg. All animals used for this study were males or females at 10–12 weeks of age. One female cohort (10–12 weeks old) was used for Neuroscore and microglia morphology analysis. Another cohort of 20-month-old male mice was used for the analysis of the Neuroscore, glutamatergic synapses and microglia analysis. All animals were housed under controlled temperature (22 °C ± 2 °C), with a 12 h light-dark cycle period and access to pelleted food and water ad libitum. All data are reported according to the ARRIVE criteria.

All surgeries were performed by the same surgeon (G.L.) at the Institute for Stroke and Dementia Research (ISD) animal facility. Only the experiment shown in Fig. EV6 was performed in Würzburg (also by G.L.), since the transgenic animals were located at the Institute for Experimental Biomedicine (Prof. D. Stegner´s lab) and could not be imported to the ISD in Munich. The only exception is the experiment shown in Fig. 3B,C and Movie EV1 that was performed in Budapest (Prof. A. Denes’ lab) by R.F.

### Transient ischemic attack (TIA) model

TIA was performed using a modification of the intraluminal filament technique as previously described (Llovera et al, 2021), animals were anesthetized (isoflurane in 30%O_2_/70%N_2_O) and received an incision between ear and eye to expose the temporal skull. A laser Doppler probe was placed over the skull above the middle cerebral artery (MCA) territory. Animals were then placed in a supine position. After a midline neck incision, the common carotid artery and left external carotid artery were isolated and ligated, a 2-mm-silicon-coated filament (Doccol, #602112PK5Re) was inserted into the internal carotid artery, through the external carotid artery, until the occlusion of the MCA, as checked by a corresponding laser Doppler flow reduction. After 5 (TIA), 15, or 30 min of MCA occlusion (MCAo), the filament was removed. For the survival period, animals were kept in their homecage with facilitated access to water and food. For 2 P imaging, a 6–0 210 um tip filament with 10-mm silicon rubber coating (#602156PK10 Doccol) was used. Sham-operated mice received the same surgical procedure, except the filament was not inserted. Predefined exclusion criterion: Mice without a reduction in blood flow to <20% of the baseline, controlled by laser Doppler flow (no animals were excluded for this criterion).

### Bilateral middle cerebral artery occlusion (BLCCAO)

Animals were anesthetized (isoflurane in 30%O_2_/70%N_2_O) and placed in a supine position. After a midline neck incision, the right and left common carotid arteries were isolated and clipped. After 5 min both clips were removed. For the survival period, animals were kept in their homecage with facilitated access to water and food.

### Cisterna magna injection for P2Y12R inhibitor and ATP

To block P2Y12R-mediated microglial actions, a P2Y12R inhibitor, PSB-0739 (#3983; Bio-Techne Corp.) dissolved in artificial cerebral spinal fluid (aCSF) (#3525, Bio-Techne Corp.; 40 mg/kg in 5 μl volume) was injected into the cisterna magna (CM) before surgery, while vehicle aCSF injection was used as a control. To mimic the focal increase of ATP, different concentrations of ATP were injected (0.3, 1.0, and 3.0 µg; Adenosine 5-triphosphate disodium salt hydrate, #A1852-1VL, Sigma) dissolved in purified water (Ambion^TM^ Nuclease-Free water, #AM9937, Invitrogen), while vehicle purified water injection was used as a control. CM injections were done under 1–1.5% isoflurane anesthesia.

### Functional outcome test

All functional tests were performed blinded

#### Neuroscore

The Neuroscore was performed before surgery, 4, 24, 48 h and 3 d after TIA; this test was used to evaluate the general status and focal neurologic dysfunction after TIA and was performed as described before (Orsini et al, 2012). The score ranges from 0 (no deficits) to 56 (representing the poorest performance in all items) and is calculated as the sum of the general and focal deficits. The Neuroscore results were expressed as a composite neurological score, which included the following general deficits (scores): fur (0–2), ears (0–2), eyes (0–4), posture (0–4), spontaneous activity (0–4), epileptic behavior (0–12); and the following focal deficits: body asymmetry (0–4), gait (0–4), climbing on a surface inclined at 45° (0–4), circling behavior (0–4), front-limb symmetry (0–4), circling behavior (0–4), and whisker response to light touch (0–4).

#### Barnes maze

A modified version described before (Attar et al, 2013) was used to perform the Barnes maze test. The elevated 20-hole apparatus (diameter: 100 cm, hole diameter: 10 cm) had a target box that was placed under the maze. The protocol includes three phases of interaction of mice with the maze: (1) habituation, (2) 3-day training, TIA surgery and 48 h later (3) probe. Before each day of training or probe, mice were placed 30 min before the procedure in the testing room for acclimatization. On day 1, mice were habituated to the maze. Therefore, mice were placed in the center of the maze in a 2-l glass beaker. After 1 min of acclimatization, mice were guided slowly by moving the glass beaker toward the target hole. On day 2, mice were placed in the center and given the possibility to freely explore and find the target hole. If mice did not reach the target hole within 2 min, the glass beaker was used to slowly guide them to the target. This was done in two consecutive trials. On days 3 and 4, mice were placed under the beaker and 10 s after placement, the beaker was removed, and mice were allowed to explore freely and find the target hole. Again, two consecutive trials were performed with every mouse. This procedure was repeated for the actual probe 3 d later. TIA surgery was performed on day 5 (after training). Acquisition and zone-dependent analysis were performed with Ethovision XT (Noldus).

### In vivo wide-field calcium imaging

To visualize calcium dynamics of cortical (L2/3) excitatory neurons, an optogenetic calcium-reporter mouse strain, C57BL/6J-Tg(Thy1- GCaMP6s)GP4.12Dkim/J (Dana et al, 2014) was used. First, an imaging window was created over the dorsal cranium as follows. The animals were anesthetized with inhalation anesthesia of isoflurane (5% for induction and 2% during operation) in 70% nitrous oxide and 30% oxygen and fixed in a prone position in a stereotaxic frame (Stoelting). Then the skin covering the skull and the underlying connective tissue were removed, and a layer of transparent dental cement (Parkell C&B Superbond) was distributed on the window area and covered with a manually fitted coverslip (Thermo Fisher). Afterward, the mice were allowed to recover from the surgery for more than 48 h before the first image acquisition.

For image acquisition, mice were injected with 0.05 mg/kg body weight of medetomidine intraperitoneally 5 min before inducing inhalation anesthesia with a mixture of 5% isoflurane in 70% nitrous oxide and 30% oxygen. After 70 s, the mice were secured in a stereotactic frame. Subsequently, the isoflurane concentration was reduced to 1.5% for 140 s, and then further decreased to 0.75% for 2 min to achieve steady state prior to data acquisition. In vivo wide-field calcium imaging was conducted using a custom-built imaging system, described by Cramer et al (2019). This system allowed mesoscale imaging of dorsal cortical brain areas across both hemispheres through the intact skull, encompassing a field of view of 10 × 10 mm. This corresponds to an image matrix resolution of 512 × 512 pixels. Image acquisition was performed over a 4-min period at a frequency of 50 Hz, capturing both fluorescent and background (no light) channels, in a room devoid of ambient light. For each subject, a total of six imaging sessions were conducted: baseline, during filament insertion, 4 h, 24 h, 3 d, and 7 d after filament insertion. Following the imaging session, anesthesia was reversed by administering Atipamezole at a dosage of 0.1 mg/kg body weight intraperitoneally. Throughout the anesthetization procedures, the body temperature of the animals was regulated using a feedback-controlled heating system. After the completion of the surgical interventions, the animals were placed in a heating chamber until full recovery from the anesthesia was observed.

Image acquisition, image processing and the calculation of functional connectivity, were conducted as previously described (Cserep et al, 2020). Briefly, subsequent to motion correction and image alignment, the background was removed from each fluorescent image. The delta F/F transformation was then applied by centering and normalizing the fluorescent images to the record-wide average for each pixel. Functional connectivity was assessed by calculating Pearson correlation coefficients for the signal time courses, followed by Fisher z-transformation, across 16 predefined regions of interest (ROIs) that represent distinct anatomical and functional brain areas. Average connectivity scores were grouped, and differences between groups were depicted for all ROI pairs using a heat map. Overall functional connectivity alterations due to TIA were evaluated by computing the global connectivity (GC) for each pixel. To assess the effects of TIA, GC scores were averaged pixel-wise within group (TIA and Sham group). The intergroup differences were then visualized using a topographical map representing all brain pixels. Network connectivity was analyzed in Matlab version 2016b, visualized in R version 4.3.1 and evaluated by repeated measures of ANOVA followed by Turkey’s post hoc test. Differences with a *p* value <0.05 were considered to be statistically significant.

### In vivo two-photon imaging

For [ATP]_e_ measurements, animals were injected with a green ATP sensor (#YL006006-AV9, AAV9-hsyn-cATP1.0(chick), WZ Biosciences, Inc., Columbia, MD, USA) 1 week before cranial window surgery. Cranial window (3 mm, circular) was placed over the primary somatosensory cortex of CX3CR1^GFP/+^or CX3CR1^tdTomato^ mice, and 2–3 weeks after surgery, [ATP]_e_ events were imaged in awake animals using the resonant-scanning light path at 960 or 920 nm with 16× water-immersion objective (Nikon CFI75 LWD 16× W, NA 0.8) at 32.7521 Hz. The acquisitions were performed before and 1 h long after TIA, the recordings typically started 5 min after TIA (5 min fMCAo). Repetitively, 2–3 min long videos (with 2 min pauses) were recorded from the same FOVs. ATP events were manually quantified for every animal. For microglial process motility measurements, the galvo light path was used at 920 nm or 1040 nm. Z-stacks (consisting of six individual planes) were recorded every minute during the 30 min baseline and 1 h after TIA sessions from anesthetized (phentanyl) mice with the 16x objective. Thirty individual microglial processes per FOV were manually tracked in 2D with FIJI, and maximum, average and median process speed were calculated according to the FIJI coordinates in Excel. Imaging was performed on a Femto2D-DualScanhead microscope (Femtonics) coupled with a Chameleon Discovery laser (Coherent). Data acquisition and analysis were performed with MESc (v.3.5.6.9395SLE) and MES (v.5.3560) software (Femtonics, Budapest, Hungary).

### [18 F]FDG positron-emission tomography (PET)

The mice were scanned before, 24 h, 3 and 7 d after the TIA induction using a 3T Mediso nanoScan PET/MR scanner (Mediso Ltd, Hungary) with a single-mouse imaging chamber. The mice received an intravenous injection of 18.0 ± 2.1 MBq[18 F]FDG through the tail vein. For the dynamic PET imaging (3TIA and 3sham mice), acquisition was performed from 0 to 90 min after tracer injection (analysis cohort). For the static PET imaging (3TIA and 3sham mice), the list-mode data were acquired at 60–90 min after tracer injection (validation cohort). A 15-min anatomical T1 MR scan was performed at 30 min after[18 F]FDG injection for the validation cohort (static imaging) and after 90 min for the analysis cohort (head receive coil, matrix size 96 ×  96 × 30, voxel size 0.21 × 0.24 × 0.65 mm^3^, repetition time 677 ms, echo time 28.56 ms, flip angle 90°). The PET field of view (FOV) included the whole mouse, while the MRI FOV covered the mouse head only. The T1 image was then used to create a body-air material map for the attenuation correction of the PET data. We reconstructed the PET list-mode data within a 400–600 keV energy window using a 3Diterative algorithm (Tera-Tomo 3D, Mediso Ltd, Hungary) with the following parameters: matrix size 55 × 62 × 187 mm^3^, voxel size 0.3 × 0.3 × 0.3 mm^3^, eight iterations, six subsets. When acquired dynamically (0–90 min p.i. acquisitions), the list-mode data were binned into 25 frames (6 × 10, 2 × 30 s, 3 × 1, 5 × 2, 5 × 5, 5 × 10min). Decay and random correction were applied.

Native space attenuation- and motion-corrected PET images were averaged and affine registered to an in-house mouse FDG-PET template and intensity normalized to the mean tracer uptake in the cerebellum. Mean intensity-normalized SUVR values were then extracted for Regions of interest included in the Mirrione atlas (Mirrione et al, 2007).

### Assessment of apoptotic cells (TUNEL)

Mice were deeply anesthetized 24 h after TIA induction with ketamine (120 mg/kg) and xylazine (16 mg/kg) and transcardially perfused with 10 ml saline. Brains were removed and frozen immediately on powdered dry ice and stored at −20 °C. About 20-µm-thick coronal sections were obtained at the level of somatosensory hindlimb (Bregma: 0.5 mm posterior). Cell death was measured with the terminal deoxynucleotidyl transferase-mediated dUTP nick-end labeling (TUNEL) method (TUNEL apoptosis detection kit; Millipore, #S7110). Finally, sections were stained with DAPI and mounted with fluoromount medium (Sigma). Two images, per animal, were taken at 40x magnification on a confocal microscope (Zeiss 880) in the striatum region, and TUNEL-positive cells were counted using FIJI software (Analyze particles).

### Tissue hypoxia staining (Hypoxyprobe)

Hypoxic tissue was detected using Hypoxyprobe-1 Green Kit (HPI, #HP6-XXX). Briefly, mice were administered with intraperitoneal injection of 60 mg/Kg Pimonidazole Hydrochloride and after 25 min mice were deeply anaesthetized with ketamine (120 mg/kg) and xylazine (16 mg/kg) and transcardially perfused with 10 ml normal saline and 10 ml 4% PFA (pH 7.4), then brains were removed and post-fixed in 4% PFA for 18 h at 4 °C. About 100-µm-thick coronal sections were obtained at the level of the somatosensory hindlimb (Bregma: 0.5 mm posterior). Brain sections were incubated with FITC-antipimonidazole Mab1 (1:100, overnight). Finally, sections were stained with DAPI and mounted with fluoromount medium (Sigma). Samples were analyzed on a confocal microscope (Zeiss 880).

### Blood–brain barrier integrity analysis

Alterations in blood–brain barrier integrity was evaluated by the Evans blue (EB) assay. Briefly, mice were administered with intraperitoneal injection of 200 µl EB solution (1% in PBS, 2.5 ml/kg; Sigma-Aldrich) and after 2 h, mice were deeply anaesthetized with ketamine (120 mg/kg) and xylazine (16 mg/kg) and transcardially perfused with 10 ml normal saline. Brains were quickly removed, weighed, minced into small pieces and incubated in 500 µl formamide (Roth) for 24 h at 55 °C. Samples were then centrifuged for 20 min at 10,000×*g*, and supernatants from each sample were transferred to a clear flat-bottom 96-well plate. The optical density of the samples was measured in duplicate on a spectrophotometer plate reader at 620 nm (Bio-Rad), and the amount of EB in the samples was quantified using the standard curve method.

### Immunohistology

Mice were transcardially perfused at the indicated time points with 10 ml saline and 10 ml 4% PFA (pH 7.4), then post-fixed in 4% PFA for 18 h at 4 °C.100-µm-thick coronal sections were obtained at the level of somatosensory hindlimb (Bregma: 0.5 mm posterior) for immune histochemical analysis. Free-floating sections were frozen in cryoprotectant and stored at −80 °C. Then sections were washed in PBS overnight at 4 °C. After washing with PBS, sections were incubated in blocking buffer containing 0.1% Triton, 0.05% Tween 20, 1% bovine serum albumin, 0.1% cold fish skin gelatine, and 2% goat or donkey serum in PBS at RT for 1 h. For the glutamatergic synapse staining, sections were incubated for 3 d at 4 °C with Guinea pig anti-VGlut1 antibody (1:1000, Millipore #AB5905), Chicken anti-Homer (1:2000, Synaptic Systems #160006) and labeled overnight at 4 °C with the secondary antibody AF488 goat anti-Guinea pig (1:500, Invitrogen), AF647 goat anti-Chicken (1:500, Invitrogen). For neuron-microglia interaction staining, sections were incubated overnight at 4 °C with rabbit anti-P2Y12 antibody (1:200, AnaspecInc#AS-55043A), mouse anti-KV2.1 (1:200, NeuroMab#75-014) and labeling for 1 h at room temperature with the secondary antibody AF488 goat anti-rabbit (1:200, Invitrogen A11034), AF647 goat anti-mouse (1:200, Invitrogen #A21235). For microglia morphology analysis staining, sections were incubated overnight at 4 °C with rabbit anti-Iba1 (1:100, Wako #019-19741) and labeling for 2 h at room temperature with the secondary antibody AF594 goat anti-rabbit (1:200, Invitrogen). For neuronal staining, sections were incubated overnight at 4 °C with mouse anti-NeuN antibody (1:100, MerkMillipore MAB377) and labeling for 1 h at room temperature with the secondary antibody AF488 goat anti-mouse (1:200, Invitrogen A11001). Finally, sections were stained with DAPI and mounted with fluoromount medium (Sigma). Two images, per animal, were taken at 40x magnification on a confocal microscope (Zeiss 880) in the cortex region, just above the hippocampus.

For the synapse analysis, confocal Z-stack images were processed, and colocalizations of VGlut and Homer were analyzed using ImageJ (synapse counter plugin).

For the microglia-synapse analysis, confocal Z-stack images were processed, and colocalizations of synapses (VGlut+ Homer+) in contact with microglial cells (P2Y12+) were analyzed using Imaris software. A 3D reconstruction for synapses (VGlut+ Homer+) and a 3D reconstruction for microglial cells (P2Y12+) were generated. Synapses in microglial cells were counted as engulfed synapses, and synapses partially in microglial cells were counted as contacts.

For the microglia–Neuron analysis, confocal Z-stack images were obtained and analyzed using ImageJ software. To measure the distribution of Kv2.1 labeling relative to microglial processes, confocal stacks were exported into single-channel TIFF series. Cell volume of eight random pyramidal neurons (KV2.1+) per image were calculated frame by frame using FIJI. Then the microglial contacts (P2y12+) to each neuron was calculated frame by frame, and the different surface contacts were calculated per each pyramidal neuron using FIJI.

For the microglia morphology analysis, confocal Z-stack images were processed, and microglial morphology features were extracted using custom-written scripts in MATLAB (R216b, The MathWorks, Natick, Massachusetts, USA), with dependencies on the Image Processing Toolbox as well as Statistics and Machine Learning Toolbox. Statistical analysis and data visualizations were performed in RStudio4 using R version 3.2.2 5 and the packages ROCR 6, plyr7, beeswarm8, and corrplot9. The detailed protocol and properties of the MATLAB script have been previously described (Heindl et al, 2018). A Kruskal-Wallis test with post hoc Bonferroni correction was applied for multilevel comparisons between groups.

### Golgi-Cox staining and dendritic spine analysis

Following saline perfusion, mice were perfused with aldehyde fixative solution (Bioenno, #003780). Brains were then carefully removed and placed in fixative solution at 4 °C overnight. Brains were then sliced at 100-μm-thick vibratome sections and immersed in impregnation solution (Bioenno, sliceGolgiKit, #003760) for 5 d. Further staining was performed as described by the manufacturer (Bioenno). In total, five dendrites from five neurons each were imaged (100× brightfield). Dendrites from the images were then reconstructed using Imaris ×64 (version 8.4.0, Bitplane).

### Cerebral open flow microperfusion (cOFM)

Brain extracellular tissue fluid was collected before and at different time points after TIA surgery using cerebral open flow microperfusion (cOFM) as previously described (Birngruber et al, 2013). Briefly, mice were anesthetized, and their head was fixed in a stereotactic frame. A 15 mm skin incision was made to expose the skull. The cOFM probe (cOFM P1-1, Joanneum research) was inserted slowly to a depth of 1 mm into the somatosensory cortex (Bregma: 1.5 lateral, 0.5 mm posterior) via a 1 mm hole drilled into the skull. The probe was fixed to the skull using a biocompatible dental cement (Quick Base S398, L-Powder clear S399, Universal Catalyst S371, Parkell C&B metabond, USA). The composition of the standard cOFM perfusate was designed to match brain extracellular fluid to avoid chemical stress. The specific composition of artificial cerebrospinal fluid (aCSF) was prepared as described in the Alzet webpage (https://www.alzet.com/guide-to-use/preparation-of-artificial-csf/): NaCl 148.18 mM; MgCl_2_ 0.8 mM (purity ≥98%); CaCl2 1.4 mM (purity ≥93%); KCl 3mM; NaH_2_PO_4_ 0.19 mM; Na_2_HPO_4_ 1.2 mM; glucose 3.7 mM; urea (Carbamide) 6.7 mM. All reagents were dissolved in sterile water (Aqua bidest; Fresenius Kabi, Graz, Austria). The perfusate was filtered through a 0.22-ml sterile filter (Thermo Fisher Scientific, Schwerte, Germany). All steps were performed under sterile conditions. Three weeks after cOFM insertion, the healing dummy was replaced by the inflow and outflow tubing before sampling; perfusate is then injected into the brain tissue and withdrawn at the same flow rate. The inflow and outflow tubing are connected to a micropump at a flow rate of 0.8 µl/min.

### Proteomics sample preparation

Brain extracellular tissue fluid was collected for 60 min using cOFM and immediately frozen on dry ice, before and at 24 h, 3 d, and 7 d after TIA.40 µl of mouse brain fluid dialysates were prepared according to the single-pot solid-phase-enhanced sample preparation (SP3) protocol (Hughes et al, 2019) with some adaptations. In brief, samples were mixed 1:1 with lysis buffer containing 10% SDS in PBS, pH 8.5, 10 mM Tris (2-carboxyethyl) phosphine, and 30 mM 2-chloroacetamide. Proteins were denatured, reduced, and alkylated by incubating samples at 70 °C, 800 rpm on a shaker for 10 min. We added 2 µl of prepared magnetic bead mixtures (Sera-Mag Magnetic carboxylate modified particles, Cytiva) to each sample and mixed them 1:1 with 100% acetonitrile (ACN). Samples were incubated for 8 min at RT and 800 rpm on a shaker. Following, samples were transferred on a magnet, and magnetic beads with bound proteins were washed two times with 200 µl 70% ethanol and once with 100% ACN while keeping the samples the whole time on the magnet. For enzymatic digestion, the magnetic beads were resuspended in 10 µl digestion buffer containing 0.1 µg trypsin/Lys-C mixture (Promega) in 50 mM ammonium bicarbonate buffer, pH 8.0 and digestion at 37 °C, 400 rpm overnight. The next day, samples were mixed with 380 µl 100% ACN and incubated for 8 min at room temperature. After transfer on a magnet, the beads were washed with 200 µl 100% ACN and air-dried. Peptides were eluted from magnetic beads by resuspending the same in 9 µl of 5% ACN in LC-MS-grade water. We determined peptide concentrations and used 250 ng peptides per sample for each LC-MS run.

### LC-MS/MS analysis

Samples were measured by liquid chromatography-tandem mass spectrometry using an Easy nLC 1200 chromatographic system (Thermo Fisher Scientific) and the Exploris 480 mass spectrometer (Thermo Fisher Scientific). Peptides were separated by 90 min chromatographic gradients using a binary buffer system with buffer A (0.1% formic acid in LC-MS-grade water) and buffer B (80% ACN, 0.1% formic acid in LC-MS-grade water). We used an in-house packed analytical column with a length of 50 cm and filled with 1.9 µm ReproSil-Pur 120 C18-AQ material (Dr. Maisch). To separate peptides, the amount of buffer B was linearly increased from 4 to 25% over 70 min with a constant flow rate of 300 nl/min. Following, buffer B was increased to 55% over 8 min and a sharp increase to 95% buffer B over 2 min. The analytical column was washed at 95% for 10 min.

Eluting peptides were ionized by nano-electrospray ionization at a constant spray voltage of 2.5 kV. We measured the samples in data-independent acquisition (DIA) mode. In brief, full MS were recorded at a resolution of 60,000 with an AGC target of 300% and a maximum injection time of 55 ms. The scan range was set to 340–1080 m/z. DIA MS/MS scans were recorded at a resolution of 15,000, an AGC target of 1000%, and a maximum injection time of 22 ms. The DIA window m/z range was set from 400–1000 m/z separated into 50 isolation windows with a size of 12 m/z per window. We used a staggered window approach with isolation windows shifted by 6 m/z every second scan cycle. Fragment ions were generated with an HCD collision energy of 27%. We measured pooled samples for the generation of a gas phase fractionation (GPF) spectral library. Similar LC-MS methods were applied, only the DIA scan ranges were limited to 100 m/z, covering the overall m/z range from 400–1000 m/z in six consecutive runs and staggered DIA windows with a size of 4 m/z. The mass spectrometry proteomics data have been deposited to the ProteomeXchange Consortium (http://proteomecentral.proteomexchange.org) via the PRIDE partner repository (https://academic.oup.com/nar/article/50/D1/D543/6415112?login=false) with the dataset identifier PXDxxxxxx.

### Statistical analysis of proteomics data

Staggered windows were deconvoluted with the MSConvert tool of the ProteoWizard software suit (v. 3.0.21321, (Chambers et al, 2012)). Spectral library generation and peptide identification/quantification from LC-MS raw data was performed with the DIA-NN software suit (v.1.8, (Demichev et al, 2020)). We used the SWISS-PROT Mus musculus fasta database downloaded from UniProt (v. 2021-11-18) to make a spectral library using the six GPF measurements from pooled samples. Trypsin was set as the digestion enzyme with a maximum of one miss-cleavage, and cysteine carbamidomethylation was set as a fixed modification. The scan window radius was set to 10, mass accuracies were fixed to 2e-05 (MS2) and 7.5e-06 (MS1), respectively. Precursor peptides were filtered at an FDR <1%. Label-free normalization of protein groups was performed in R using the MaxLFQ algorithm (Cox et al, 2014) and proteotypic peptides only.

Statistical analysis of the data were performed with the Perseus software suite (v. 1.6.15 (Tyanova et al, 2016)). In brief, we log2-transformed protein LFQ intensities and filtered proteins for data completeness in at least one time point. Missing values were replaced sample-wise by random drawing of numbers from 1.8 standard deviations downshifted, and 0.3 standard deviations broad normal distributions. Quantile normalization was performed with an R Perseus-plugin as well as a batch correction with ComBat R Perseus-plugin (batch = animal). Significantly regulated proteins were identified by ANOVA multiple sample testing (S0 = 0.1, permutation-based FDR = 0.05, 250 randomizations). ANOVA-significant proteins were further *Z*-score normalized and used for hierarchical clustering using Euclidean distances. We searched systematically for enriched processes in identified clusters using Fisher’s exact testing (Benjamini–Hochberg FDR = 0.02).

### ATP luciferase assay

ATP concentration was determined using a luciferase assay as previously described (Sebastian-Serrano et al, 2018). Brain extracellular tissue fluid was collected for 15 min using cOFM and placed in a tube with 1 µl of 100 mM ARL 67156 (ARL 67156 trisodium salt hydrate, #A256-5MG, Sigma), a competitive inhibitor of ecto-ATPases and immediately frozen on dry ice to further determine the ATP concentration. The nucleotide concentration in the brain extracellular tissue fluid was measured using ENLITENR rLuciferase/Luciferin reagent (#FF2021, Promega) according to the manufacturer’s protocol. Briefly, 2 µl of brain extracellular tissue fluid was transferred to wells of a 96-well plate placed on ice. The 96-well plate was set in a Glomax-multi Microplate Luminometer (Promega GmbH), and 100 mL of rLuciferase/Luciferin reagent was automatically injected into each well at room temperature.

### Cell isolation for RNA sequencing and loading Chromium Next GEM Chip G

Mice were perfused with ice-cold saline, and brains were gently removed. The hemispheres were split, and the white matter and meninges were removed with forceps. The tissue was dissociated using the papain dissociation system (#LK003150, Worthington Biochemical Corporation) according to the manufacturer’s protocol and an incubation time of 15 min. The cell suspension was cleared from dead cells and cell debris using the dead cell removing kit (#130-090-101, Miltenyi). Propidium iodide-negative live single cells were flow cytometrically sorted using a BD Fusion cell sorter into RPMI medium containing 5% fetal bovine serum. The cell suspension was centrifuged and washed with PBS containing 0.04% bovine serum albumin (BSA). The cells were resuspended in PBS containing 0.04% BSA for a concentration of 1200 cells/µl, counted and loaded into the Chromium Next GEM Chip G (PN-1000127, 10x Genomics) for a target recovery of 10,000cells according to the 10x Genomics Next GEM Single Cell 3‘ v3.1 (Dual Index) kit protocol (Step 1, CG000315, Rev E) for GEM preparation (PN-1000296, 10x Genomics) and run in a 10x Genomics Chromium Controller. After GEM preparation, the protocol of the kit was followed for post-GEM-RT Cleanup & cDNA amplification (Step 2) and 3′ gene expression library construction (Step 3). Single-cell libraries were sequenced using an Illumina HiSeq 1500 sequencer with a sequencing depth of >20,000 reads per cell.

### Single-cell transcriptomic data analysis

Samples were demultiplexed, processed and aligned to the reference genome (mm10, GENCODE vM23/Ensembl 98), and unique molecular identifier (UMI) counts were summarized using CellRanger software (10x Genomics, v. 7.1.0). Filtered gene-barcode matrices were used for further analysis and processed using the Seurat package in R, version 4.3.0 (Hao et al, 2021). Cells that expressed fewer than 500 genes and contained more than 7% of mitochondrial genes were excluded, and genes expressed in less than three cells were removed from the data count matrices. After quality control, the data were log-normalized, and variable features were identified using the *vst* method. Scaling and regression against the number of UMIs and mitochondrial RNA content per cell was applied. Unbiased clustering was applied using k-nearest neighbor calculation, and dimensionality reduction was performed using PCA and UMAP. Differentially expressed genes among the clusters were identified using the FindAllMarkers function of the Seurat package. Figure 2E shows absolute gene counts of the preprocessed scRNAseq dataset per cell group for time points 24 h and 3 d after TIA induction compared to the control condition. Since the differential gene expression analysis was performed as pseudo-bulk using the function “FindAllMarkers()” in Seurat, which uses average expression over all cells.

Differentially regulated genes per cell type (Fig. EV4A) were calculated from log-normalized expression values from raw gene counts, which were then log-normalized and scaled (*z*-score). For the barplot, all genes with a *p* value <0.05 for differential expression were included. Gene set enrichment analysis (GSEA) was performed based on these significantly differentially regulated genes using ClusterProfiler (Version 4.7.2). All analysis scripts will be made available upon publication.

### Microglia transcriptomics (Nanostring)

Mice were perfused transcardially with ice-cold saline containing Heparin (2 U/mL). Brains were placed in HBSS (w/divalent cations Ca^2+^ and Mg^2+^) supplemented with actinomycin D (1:1000, 1 mg/mL, Sigma, #A1410), and microglia was isolated with the Papain-based Neural Tissue Dissociation Kit (P) (# 130-092-628, MiltenyiBiotec B.V. & Co. KG) according to the manufacturer’s instructions. Cell suspension was enriched using a 30% isotonic Percoll gradient. 1 × 10^3^–1.5 × 10^3^ live microglia-like cells from three mice per condition were sorted according to their surface marker CD45 + CD11b + 7-AAD negative (SH800S Cell Sorter, Sony Biotechnology). Microglia-like cells' total RNA was extracted using the Arcturus PicoPure RNA Isolation Kit (Applied Biosystems #KIT0204). About 65 ng of total RNA per sample was then hybridized with reporter and capture probes for Counter Gene Expression code sets (Mouse Neuroinflammation code set) according to the manufacturer’s instructions (NanoString Technologies). Samples were injected into the NanoString cartridge and a measurement run was performed according to the nCounter SPRINT protocol. Background (negative control) was quantified by code set intrinsic molecular color-coded barcodes lacking the RNA linkage. As a positive control code set, intrinsic control RNAs were used at increasing concentrations. Genes below the maximal values of the negative controls were excluded from the analysis. Data was analyzed by ROSALIND® (https://rosalind.bio/), with a HyperScale architecture developed by ROSALIND, Inc. (San Diego, CA). Read Distribution percentages, violin plots, identity heatmaps, and sample MDS plots were generated as part of the QC step. Normalization, fold changes and *p* values were calculated using criteria provided by Nanostring. ROSALIND® follows the nCounter® Advanced Analysis protocol of dividing counts within a lane by the geometric mean of the normalizer probes from the same lane. Housekeeping probes to be used for normalization are selected based on the geNorm algorithm as implemented in the NormqPCR R library (Perkins et al, 2012). Abundance of various cell populations is calculated on ROSALIND using the Nanostring Cell Type Profiling Module. ROSALIND performs a filtering of Cell Type Profiling results to include results that have scores with a *p* value greater than or equal to 0.05. Fold changes and *p* values are calculated using the fast method as described in the nCounter® Advanced Analysis 2.0 User Manual. *P* value adjustment is performed using the Benjamini–Hochberg method of estimating false discovery rates (FDR). Significantly regulated genes were identified by ANOVA multiple sample testing (S0 = 0.1, permutation-based FDR = 0.05). ANOVA-significant genes were further *Z*-score normalized and used for hierarchical clustering using Euclidean distances, using the Next Generation Clustered Heat Map (NG-CHM) software (GUI Builder 2.22.0 w/NG-CHM 2.24.0-build-20)(Ryan et al, 2019). We searched systematically for enriched processes in identified clusters using Fisher’s exact testing (Benjamini–Hochberg FDR = 0.05). Further, we performed enrichment analyses (Benjamini–Hochberg FDR = 0.05) on fold changes calculated for binary comparisons(Kolberg et al, 2023).

### Platelet assays

For platelet isolation, anesthetized mice underwent blood collection via insertion of a glass capillary into the retro-orbital venous plexus. Blood was collected into tubes containing 1/7 volume of acid-citrate-dextrose (ACD; 39 mM citric acid, 75 mM sodium citrate, and 135 mM dextrose) and immediately diluted 1:1 with modified Tyrode’s buffer (137 mM NaCl, 2.8 mM KCl, 12 mM NaHCO₃, 5.5 mM sucrose, 10 mM HEPES, and pH 6.5). Samples were centrifuged at 70×*g* for 15 min to obtain platelet-rich plasma (PRP). For preparation of washed platelets, PRP was diluted 1:2 in modified Tyrode’s buffer supplemented with prostacyclin (PGI₂, 0.1 mg/ml, Abcam) and casein (0.01%, Sigma), followed by centrifugation at 1000×g for 5–10 min. The platelet pellet was resuspended in Tyrode’s buffer, and platelet counts were determined using a Sysmex XN-V Series XN-1000V cell counter.

For platelet aggregation, optical aggregometry was performed as previously described by our collaborators (Nicolai et al, 2020). Briefly, washed murine platelets were adjusted to a final concentration of 2 × 10⁵/µl in modified Tyrode’s buffer. Platelet activation was induced by the addition of adenosine diphosphate (ADP; 20 µM, Sigma, #01905) targeting the P2Y12 receptor, together with calcium chloride (2 mM), under continuous stirring at 1000 rpm at 37 °C. Aggregation was recorded using a two-channel aggregometer (ChronoLog490-2D, Havertown, USA), and the maximal aggregation response was analyzed 6 min after agonist addition using Aggrolink software (ChronoLog, USA).

Platelet activation in suspension was assessed as previously described by our collaborators (Nicolai et al, 2020). Washed murine platelets (2 × 10⁵/µl) were diluted 1:10 in modified Tyrode’s buffer, activated by the addition of calcium (1 mM) and ADP (20 µM), and incubated for 30 min at room temperature with fluorescently labeled antibodies targeting the activation markers P-selectin (JonA-PE, #D200, Emfret Analytics) and activated GPIIbIIIa (CD62P-PE/Cy7, #148310, BioLegend). Following activation, platelets were fixed with 1% paraformaldehyde (PFA, Sigma) for 10 min in the dark. Samples were analyzed using a BD LSRFortessa flow cytometer, and subpopulation gating as well as mean fluorescence intensity (MFI) analyses were performed using FlowJo software (version 10).

### Statistical analysis

Data were analyzed using GraphPad Prism version 6.0. Summary data are expressed as the mean ± standard deviation (SD) for all datasets. All datasets were tested for normality using the Shapiro–Wilk normality test. The groups containing normally distributed data were tested using a two-way Student’s *t*-test (for two groups) or ANOVA (for >2 groups). The remaining data were analyzed using the Mann–Whitney *U*-test. Neuroscore data were treated as non-parametric data. Two-way ANOVA, corrected for multiple comparisons using two-stage step-up method of Benjamin Kriegel and Yekutielior Mann–Whitney *U*-test have been used in the analysis. Differences with a *p* value <0.05 were considered to be statistically significant.

## Supplementary information

Movie EV1

Peer Review File

Source data Fig. 1

Source data Fig. 2

Source data Fig. 3

Figure EV Source Data

Expanded View Figures

## Data Availability

The datasets and computer code produced in this study are available in the following databases: scRNAseq data: Gene Expression Omnibus GSE272954. Nanostring data: Gene Expression Omnibus GSE312513. Proteomics data: ProteomeXchange PXD070754. Immunofluorescent images: Bioimage archive S-BIAD2496 (Fig. 1E), S-BIAD2497 (Fig. 1F), S-BIAD2498 (Fig. 2H), S-BIAD2499 (Fig. 2I), S-BIAD2500 (Fig. 3D), S-BIAD2501 (Fig. 3E), S-BIAD2502 (Fig. 3F), S-BIAD2503 (Fig. 3G), S-BIAD2504 (Fig. 3H). The source data of this paper are collected in the following database record: biostudies:S-SCDT-10_1038-S44321-026-00397-6.

## References

[CR1] Attar A, Liu TY, Chan WTC, Hayes J, Nejad M, Lei KC, Bitan G (2013) A shortened Barnes maze protocol reveals memory deficits at 4-months of age in the triple-transgenic mouse model of Alzheimer’s disease. PLoS ONE 8:e80355 24236177 10.1371/journal.pone.0080355 PMC3827415

[CR2] Berki P, Cserep C, Kornyei Z, Posfai B, Szabadits E, Domonkos A, Kellermayer A, Nyerges M, Wei X, Mody I et al (2024) Microglia contribute to neuronal synchrony despite endogenous ATP-related phenotypic transformation in acute mouse brain slices. Nat Commun 15:5402 38926390 10.1038/s41467-024-49773-1 PMC11208608

[CR3] Birngruber T, Ghosh A, Perez-Yarza V, Kroath T, Ratzer M, Pieber TR, Sinner F (2013) Cerebral open flow microperfusion: a new in vivo technique for continuous measurement of substance transport across the intact blood-brain barrier. Clin Exp Pharmacol Physiol 40:864–871 24256164 10.1111/1440-1681.12174

[CR4] Block ML, Zecca L, Hong JS (2007) Microglia-mediated neurotoxicity: uncovering the molecular mechanisms. Nat Rev Neurosci 8:57–69 17180163 10.1038/nrn2038

[CR5] Brown RD Jr, Petty GW, O’Fallon WM, Wiebers DO, Whisnant JP (1998) Incidence of transient ischemic attack in Rochester, Minnesota, 1985-1989. Stroke 29:2109–2113 9756590 10.1161/01.str.29.10.2109

[CR6] Cancelli I, Janes F, Gigli GL, Perelli A, Zanchettin B, Canal G, D’Anna L, Russo V, Barbone F, Valente M (2011) Incidence of transient ischemic attack and early stroke risk: validation of the ABCD2 score in an Italian population-based study. Stroke 42:2751–2757 21836095 10.1161/STROKEAHA.110.612705

[CR7] Chambers MC, Maclean B, Burke R, Amodei D, Ruderman DL, Neumann S, Gatto L, Fischer B, Pratt B, Egertson J et al (2012) A cross-platform toolkit for mass spectrometry and proteomics. Nat Biotechnol 30:918–920 23051804 10.1038/nbt.2377 PMC3471674

[CR8] Chen Y, Luan P, Liu J, Wei Y, Wang C, Wu R, Wu Z, Jing M (2024) Spatiotemporally selective astrocytic ATP dynamics encode injury information sensed by microglia following brain injury in mice. Nat Neurosci 27:1522–1533 10.1038/s41593-024-01680-w 38862791

[CR9] Cox J, Hein MY, Luber CA, Paron I, Nagaraj N, Mann M (2014) Accurate proteome-wide label-free quantification by delayed normalization and maximal peptide ratio extraction, termed MaxLFQ. Mol Cell Proteomics 13:2513–2526 24942700 10.1074/mcp.M113.031591 PMC4159666

[CR10] Cramer JV, Gesierich B, Roth S, Dichgans M, During M, Liesz A (2019) In vivo widefield calcium imaging of the mouse cortex for analysis of network connectivity in health and brain disease. Neuroimage 199:570–584 31181333 10.1016/j.neuroimage.2019.06.014

[CR11] Cserep C, Posfai B, Lenart N, Fekete R, Laszlo ZI, Lele Z, Orsolits B, Molnar G, Heindl S, Schwarcz AD et al (2020) Microglia monitor and protect neuronal function through specialized somatic purinergic junctions. Science 367:528–537 31831638 10.1126/science.aax6752

[CR12] Cserep C, Schwarcz AD, Posfai B, Laszlo ZI, Kellermayer A, Kornyei Z, Kisfali M, Nyerges M, Lele Z, Katona I et al (2022) Microglial control of neuronal development via somatic purinergic junctions. Cell Rep 40:111369 36130488 10.1016/j.celrep.2022.111369 PMC9513806

[CR13] Dana H, Chen TW, Hu A, Shields BC, Guo C, Looger LL, Kim DS, Svoboda K (2014) Thy1-GCaMP6 transgenic mice for neuronal population imaging in vivo. PLoS ONE 9:e108697 25250714 10.1371/journal.pone.0108697 PMC4177405

[CR14] Davalos D, Grutzendler J, Yang G, Kim JV, Zuo Y, Jung S, Littman DR, Dustin ML, Gan WB (2005) ATP mediates rapid microglial response to local brain injury in vivo. Nat Neurosci 8:752–758 15895084 10.1038/nn1472

[CR15] Degan D, Ornello R, Tiseo C, De Santis F, Pistoia F, Carolei A, Sacco S (2017) Epidemiology of transient ischemic attacks using time- or tissue-based definitions: a population-based study. Stroke 48:530–536 28143922 10.1161/STROKEAHA.116.015417

[CR16] Demichev V, Messner CB, Vernardis SI, Lilley KS, Ralser M (2020) DIA-NN: neural networks and interference correction enable deep proteome coverage in high throughput. Nat Methods 17:41–44 31768060 10.1038/s41592-019-0638-x PMC6949130

[CR17] Denstaedt SJ, Spencer-Segal JL, Newstead MW, Laborc K, Zhao AP, Hjelmaas A, Zeng X, Akil H, Standiford TJ, Singer BH (2018) S100A8/A9 drives neuroinflammatory priming and protects against anxiety-like behavior after sepsis. J Immunol 200:3188–3200 29563178 10.4049/jimmunol.1700834 PMC5915914

[CR18] Easton JD, Saver JL, Albers GW, Alberts MJ, Chaturvedi S, Feldmann E, Hatsukami TS, Higashida RT, Johnston SC, Kidwell CS et al (2009) Definition and evaluation of transient ischemic attack: a scientific statement for healthcare professionals from the American Heart Association/American Stroke Association Stroke Council; Council on Cardiovascular Surgery and Anesthesia; Council on Cardiovascular Radiology and Intervention; Council on Cardiovascular Nursing; and the Interdisciplinary Council on Peripheral Vascular Disease. The American Academy of Neurology affirms the value of this statement as an educational tool for neurologists. Stroke 40:2276–2293 19423857 10.1161/STROKEAHA.108.192218

[CR19] Fasth O, Lesen E, Appelros P, Farahmand B, Hedberg J, Ladenvall P, Mellstrom C, Asberg S (2021) Age in relation to comorbidity and outcome in patients with high-risk TIA or minor ischemic stroke: a Swedish national observational study. Eur Stroke J 6:53–61 33817335 10.1177/2396987320975980 PMC7995324

[CR20] Gattellari M, Goumas C, Garden F, Worthington JM (2012) Relative survival after transient ischaemic attack: results from the Program of Research Informing Stroke Management (PRISM) study. Stroke 43:79–85 22076008 10.1161/STROKEAHA.111.636233

[CR21] Giles MF, Rothwell PM (2007) Risk of stroke early after transient ischaemic attack: a systematic review and meta-analysis. Lancet Neurol 6:1063–1072 17993293 10.1016/S1474-4422(07)70274-0

[CR22] Hao Y, Hao S, Andersen-Nissen E, Mauck 3rdWM, Zheng S, Butler A, Lee MJ, Wilk AJ, Darby C, Zager M et al (2021) Integrated analysis of multimodal single-cell data. Cell 184:3573–3587.e3529 34062119 10.1016/j.cell.2021.04.048 PMC8238499

[CR23] Haynes SE, Hollopeter G, Yang G, Kurpius D, Dailey ME, Gan WB, Julius D (2006) The P2Y12 receptor regulates microglial activation by extracellular nucleotides. Nat Neurosci 9:1512–1519 17115040 10.1038/nn1805

[CR24] Heindl S, Gesierich B, Benakis C, Llovera G, Duering M, Liesz A (2018) Automated morphological analysis of microglia after stroke. Front Cell Neurosci 12:106 29725290 10.3389/fncel.2018.00106 PMC5917008

[CR25] Hill MD, Yiannakoulias N, Jeerakathil T, Tu JV, Svenson LW, Schopflocher DP (2004) The high risk of stroke immediately after transient ischemic attack: a population-based study. Neurology 62:2015–2020 15184607 10.1212/01.wnl.0000129482.70315.2f

[CR26] Hong S, Beja-Glasser VF, Nfonoyim BM, Frouin A, Li S, Ramakrishnan S, Merry KM, Shi Q, Rosenthal A, Barres BA et al (2016) Complement and microglia mediate early synapse loss in Alzheimer mouse models. Science 352:712–716 27033548 10.1126/science.aad8373 PMC5094372

[CR27] Horvath G, Goloncser F, Csolle C, Kiraly K, Ando RD, Baranyi M, Kovanyi B, Mate Z, Hoffmann K, Algaier I et al (2014) Central P2Y12 receptor blockade alleviates inflammatory and neuropathic pain and cytokine production in rodents. Neurobiol Dis 70:162–178 24971933 10.1016/j.nbd.2014.06.011 PMC4148180

[CR28] Huang JM, Zhao N, Hao XN, Li SY, Wei D, Pu N, Peng GH, Tao Y (2024) CX3CL1/CX3CR1 signaling mediated neuroglia activation is implicated in the retinal degeneration: a potential therapeutic target to prevent photoreceptor death. Invest Ophthalmol Vis Sci 65:29 38231527 10.1167/iovs.65.1.29 PMC10795588

[CR29] Hughes CS, Moggridge S, Muller T, Sorensen PH, Morin GB, Krijgsveld J (2019) Single-pot, solid-phase-enhanced sample preparation for proteomics experiments. Nat Protoc 14:68–85 30464214 10.1038/s41596-018-0082-x

[CR30] Hurford R, Li L, Lovett N, Kubiak M, Kuker W, Rothwell PM, Oxford Vascular S (2019) Prognostic value of “tissue-based” definitions of TIA and minor stroke: population-based study. Neurology 92:e2455–e2461 30996061 10.1212/WNL.0000000000007531 PMC6541432

[CR31] Ippen FM, Walter F, Hametner C, Gumbinger C, Nagel S, Purrucker JC, Mundiyanapurath S (2021) Age-dependent differences in the rate and symptoms of TIA mimics in patients presenting with a suspected TIA to a neurological emergency room. Front Neurol 12:644223 33658979 10.3389/fneur.2021.644223 PMC7917180

[CR32] Johnston SC, Easton JD, Farrant M, Barsan W, Conwit RA, Elm JJ, Kim AS, Lindblad AS, Palesch YY, Clinical Research Collaboration NETTN et al (2018) Clopidogrel and aspirin in acute ischemic stroke and high-risk TIA. N Engl J Med 379:215–225 29766750 10.1056/NEJMoa1800410 PMC6193486

[CR33] Khare S (2016) Risk factors of transient ischemic attack: an overview. J Midlife Health 7:2–7 27134474 10.4103/0976-7800.179166 PMC4832890

[CR34] Kolberg L, Raudvere U, Kuzmin I, Adler P, Vilo J, Peterson H (2023) g:Profiler-interoperable web service for functional enrichment analysis and gene identifier mapping (2023 update). Nucleic Acids Res 51:W207–W212 37144459 10.1093/nar/gkad347 PMC10320099

[CR35] Llovera G, Simats A, Liesz A (2021) Modeling stroke in mice: transient middle cerebral artery occlusion via the external carotid artery. J Vis Exp (171): e62573. 10.3791/62573 10.3791/62573 34096920

[CR36] Luengo-Fernandez R, Paul NL, Gray AM, Pendlebury ST, Bull LM, Welch SJ, Cuthbertson FC, Rothwell PM, Oxford Vascular S (2013) Population-based study of disability and institutionalization after transient ischemic attack and stroke: 10-year results of the Oxford Vascular Study. Stroke 44:2854–2861 23920019 10.1161/STROKEAHA.113.001584 PMC4946627

[CR37] Mirrione MM, Schiffer WK, Fowler JS, Alexoff DL, Dewey SL, Tsirka SE (2007) A novel approach for imaging brain-behavior relationships in mice reveals unexpected metabolic patterns during seizures in the absence of tissue plasminogen activator. Neuroimage 38:34–42 17707126 10.1016/j.neuroimage.2007.06.032 PMC2084071

[CR38] Navis A, Garcia-Santibanez R, Skliut M (2019) Epidemiology and outcomes of ischemic stroke and transient ischemic attack in the adult and geriatric population. J Stroke Cerebrovasc Dis 28:84–89 30297169 10.1016/j.jstrokecerebrovasdis.2018.09.013

[CR39] Nicolai L, Schiefelbein K, Lipsky S, Leunig A, Hoffknecht M, Pekayvaz K, Raude B, Marx C, Ehrlich A, Pircher J et al (2020) Vascular surveillance by haptotactic blood platelets in inflammation and infection. Nat Commun 11:5778 33188196 10.1038/s41467-020-19515-0 PMC7666582

[CR40] Orsini F, Villa P, Parrella S, Zangari R, Zanier ER, Gesuete R, Stravalaci M, Fumagalli S, Ottria R, Reina JJ et al (2012) Targeting mannose-binding lectin confers long-lasting protection with a surprisingly wide therapeutic window in cerebral ischemia. Circulation 126:1484–1494 22879370 10.1161/CIRCULATIONAHA.112.103051 PMC3478764

[CR41] Pedrono E, Durukan A, Strbian D, Marinkovic I, Shekhar S, Pitkonen M, Abo-Ramadan U, Tatlisumak T (2010) An optimized mouse model for transient ischemic attack. J Neuropathol Exp Neurol 69:188–195 20084015 10.1097/NEN.0b013e3181cd331c

[CR42] Pendlebury ST, Rothwell PM, Oxford Vascular S (2019) Incidence and prevalence of dementia associated with transient ischaemic attack and stroke: analysis of the population-based Oxford Vascular Study. Lancet Neurol 18:248–258 30784556 10.1016/S1474-4422(18)30442-3 PMC6390174

[CR43] Perez-Sisques L, Sancho-Balsells A, Solana-Balaguer J, Campoy-Campos G, Vives-Isern M, Soler-Palazon F, Anglada-Huguet M, Lopez-Toledano MA, Mandelkow EM, Alberch J et al (2021) RTP801/REDD1 contributes to neuroinflammation severity and memory impairments in Alzheimer’s disease. Cell Death Dis 12:616 34131105 10.1038/s41419-021-03899-y PMC8206344

[CR44] Perkins JR, Dawes JM, McMahon SB, Bennett DL, Orengo C, Kohl M (2012) ReadqPCR and NormqPCR: R packages for the reading, quality checking and normalisation of RT-qPCR quantification cycle (Cq) data. BMC Genomics 13:296 22748112 10.1186/1471-2164-13-296 PMC3443438

[CR45] Perry JJ, Yadav K, Syed S, Shamy M (2022) Transient ischemic attack and minor stroke: diagnosis, risk stratification and management. CMAJ194:E1344–E1349 36220167 10.1503/cmaj.220344 PMC9616153

[CR46] Powers WJ, Rabinstein AA, Ackerson T, Adeoye OM, Bambakidis NC, Becker K, Biller J, Brown M, Demaerschalk BM, Hoh B et al (2019) Guidelines for the early management of patients with acute ischemic stroke: 2019 update to the 2018 guidelines for the early management of acute ischemic stroke: a guideline for healthcare professionals from the American Heart Association/American Stroke Association. Stroke 50:e344–e418 31662037 10.1161/STR.0000000000000211

[CR47] Quenault A, Martinez de Lizarrondo S, Etard O, Gauberti M, Orset C, Haelewyn B, Segal HC, Rothwell PM, Vivien D, Touze E et al (2017) Molecular magnetic resonance imaging discloses endothelial activation after transient ischaemic attack. Brain 140:146–157 28031221 10.1093/brain/aww260 PMC5226059

[CR48] Rodrigues RJ, Tome AR, Cunha RA (2015) ATP as a multi-target danger signal in the brain. Front Neurosci 9:148 25972780 10.3389/fnins.2015.00148 PMC4412015

[CR49] Ryan MC, Stucky M, Wakefield C, Melott JM, Akbani R, Weinstein JN, Broom BM (2019) Interactive clustered heat map builder: an easy web-based tool for creating sophisticated clustered heat maps. F1000Res 8:1750 10.12688/f1000research.20590.1 PMC7111501 32269754

[CR50] Sebastian-Serrano A, de Diego-Garcia L, Henshall DC, Engel T, Diaz-Hernandez M (2018) Haploinsufficient TNAP mice display decreased extracellular ATP levels and expression of Pannexin-1 channels. Front Pharmacol 9:170 29551976 10.3389/fphar.2018.00170 PMC5841270

[CR51] Sivakumar L, Camicioli R, Butcher K (2014) Factors associated with cognitive decline in transient ischemic attack patients. Can J Neurol Sci 41:303–313 24718815 10.1017/s0317167100017248

[CR52] Stegner D, Deppermann C, Kraft P, Morowski M, Kleinschnitz C, Stoll G, Nieswandt B (2013) Munc13-4-mediated secretion is essential for infarct progression but not intracranial hemostasis in acute stroke. J Thromb Haemost 11:1430–1433 23659589 10.1111/jth.12293

[CR53] Touze E, Varenne O, Chatellier G, Peyrard S, Rothwell PM, Mas JL (2005) Risk of myocardial infarction and vascular death after transient ischemic attack and ischemic stroke: a systematic review and meta-analysis. Stroke 36:2748–2755 16254218 10.1161/01.STR.0000190118.02275.33

[CR54] Tyanova S, Temu T, Sinitcyn P, Carlson A, Hein MY, Geiger T, Mann M, Cox J (2016) The Perseus computational platform for comprehensive analysis of (prote)omics data. Nat Methods 13:731–740 27348712 10.1038/nmeth.3901

[CR55] Voss OH, Tian L, Murakami Y, Coligan JE, Krzewski K (2015) Emerging role of CD300 receptors in regulating myeloid cell efferocytosis. Mol Cell Oncol 2:e964625 27308512 10.4161/23723548.2014.964625 PMC4905414

[CR56] Wang J, Zhang P, Tang Z (2020) Animal models of transient ischemic attack: a review. Acta Neurol Belg 120:267–275 32048230 10.1007/s13760-020-01295-5 PMC7083805

[CR57] Wang Y, Johnston SC, Wang Y (2013) Clopidogrel with aspirin in minor stroke or transient ischemic attack. N Engl J Med 369:1376–1377 24088101 10.1056/NEJMc1309713

[CR58] Wu CM, McLaughlin K, Lorenzetti DL, Hill MD, Manns BJ, Ghali WA (2007) Early risk of stroke after transient ischemic attack: a systematic review and meta-analysis. Arch Intern Med 167:2417–2422 18071162 10.1001/archinte.167.22.2417

