# Supplementary material for: Blocking microglial reactivity via purinergic receptors prevents subacute cognitive deficits after TIA
Source: EMBO Mol Med. 2026 Mar 20;18(4):1150–73. doi: 10.1038/s44321-026-00397-6 (PMC13083932; doi:10.1038/s44321-026-00397-6)
Supplement: Supplementary file 1 — Movie EV1 [file 44321_2026_397_MOESM1_ESM.zip › Movie EV1/Movie EV1 legend.docx]

**Movie EV1**. Microglial processes (Cx3Cr1cre-TdTomato -red-) recruitment to focal increase in extracellular ATP (hsyn-cATP -green-) within 3min after TIA
