# Supplementary figures and images for: Blocking microglial reactivity via purinergic receptors prevents subacute cognitive deficits after TIA
Source: EMBO Mol Med. 2026 Mar 20;18(4):1150–73. doi: 10.1038/s44321-026-00397-6 (PMC13083932; doi:10.1038/s44321-026-00397-6)

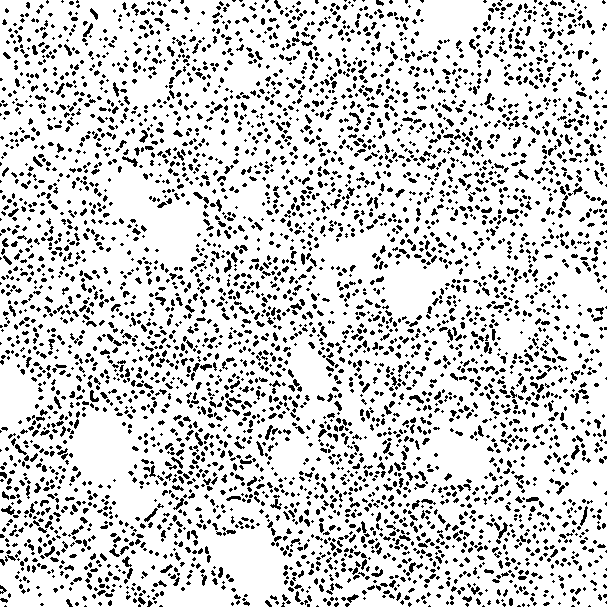

Supplement: Supplementary file 3 — Source data Fig. 1 [file 44321_2026_397_MOESM3_ESM.zip › Figure 1/Figure1F/Fig1Fb_original.tiff]

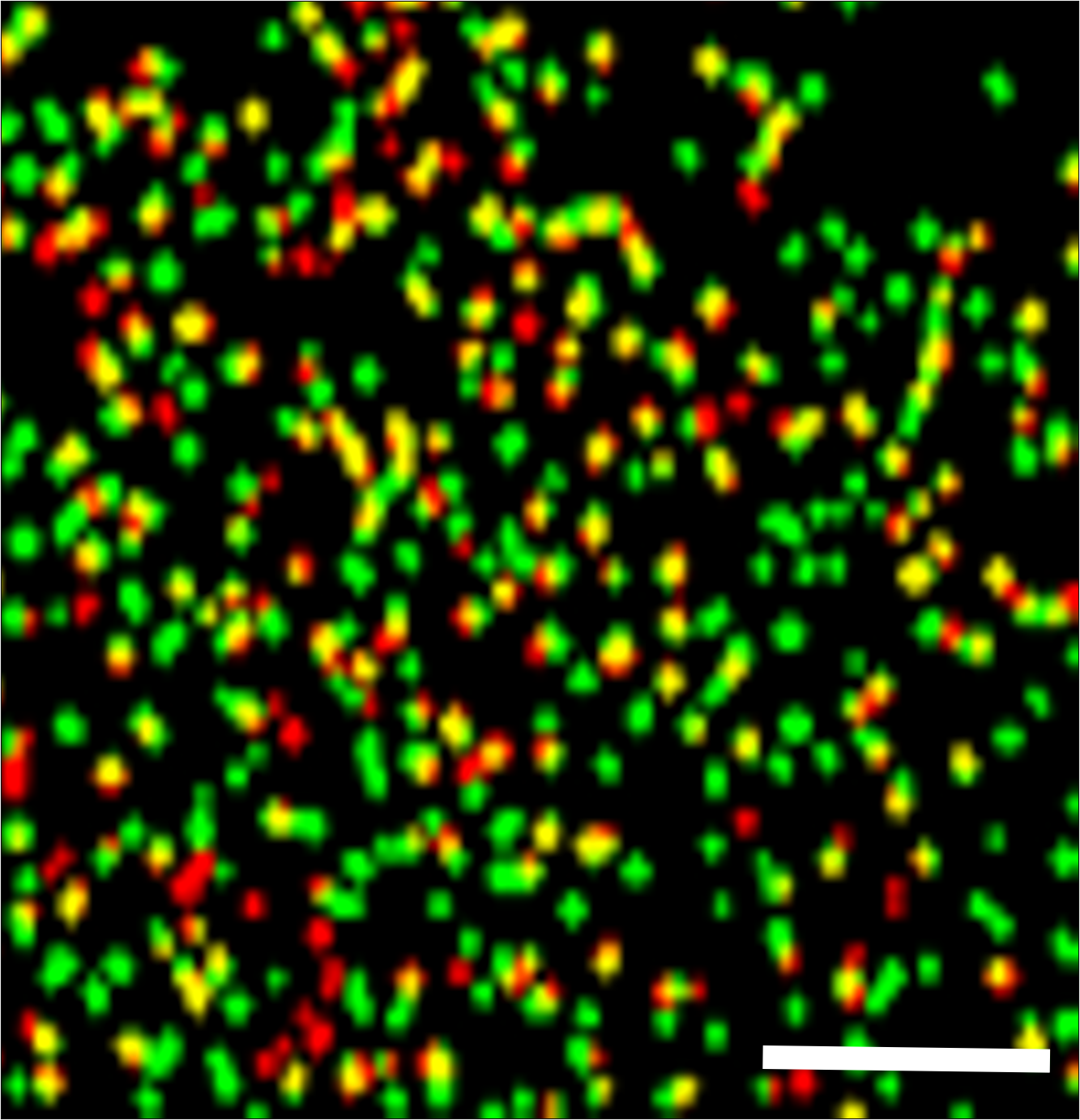

Supplement: Supplementary file 3 — Source data Fig. 1 [file 44321_2026_397_MOESM3_ESM.zip › Figure 1/Figure1F/Fig1F.tif]

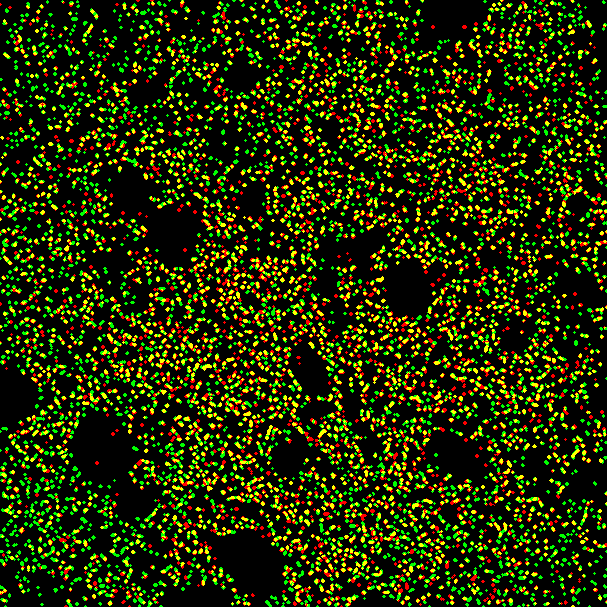

Supplement: Supplementary file 3 — Source data Fig. 1 [file 44321_2026_397_MOESM3_ESM.zip › Figure 1/Figure1F/Fig1F_original.tif]

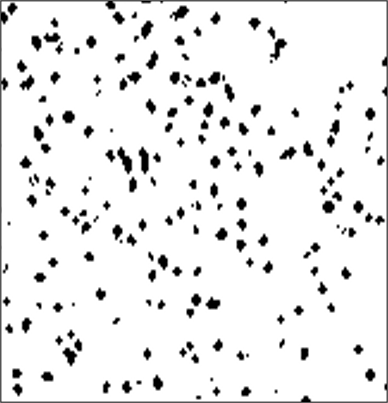

Supplement: Supplementary file 3 — Source data Fig. 1 [file 44321_2026_397_MOESM3_ESM.zip › Figure 1/Figure1F/Fig1Fb.tif]

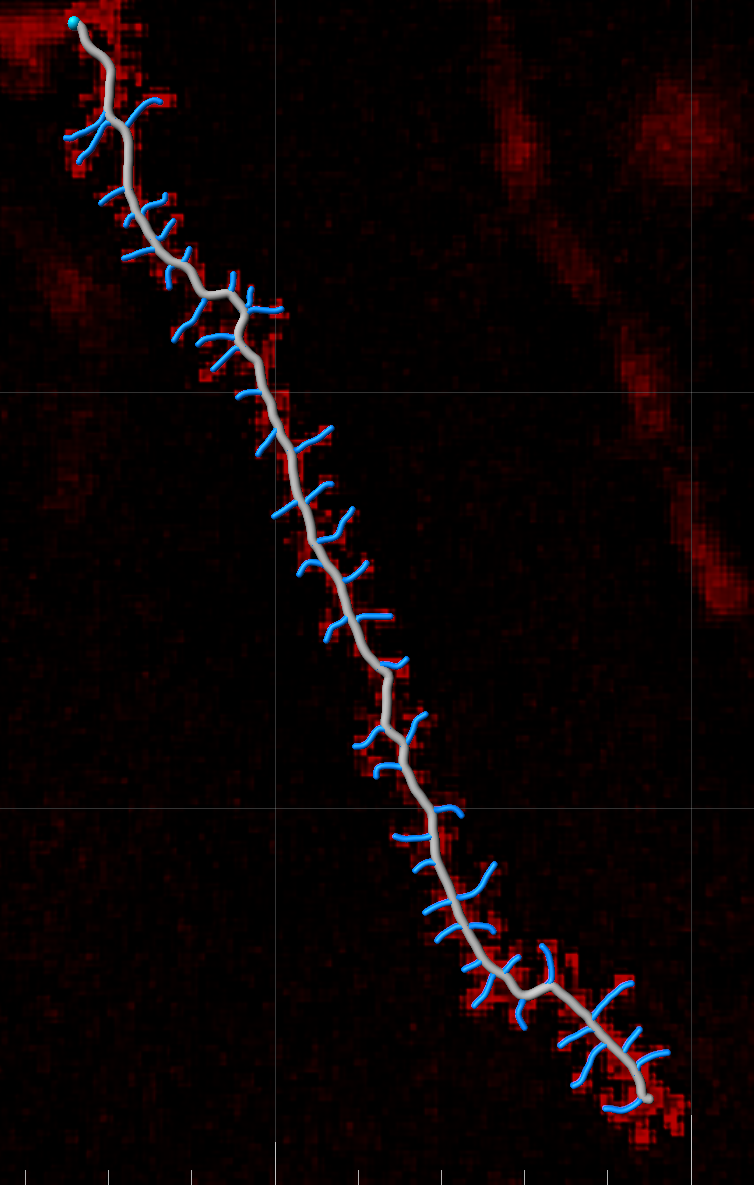

Supplement: Supplementary file 3 — Source data Fig. 1 [file 44321_2026_397_MOESM3_ESM.zip › Figure 1/Figure1E/Figure1E_small.png]

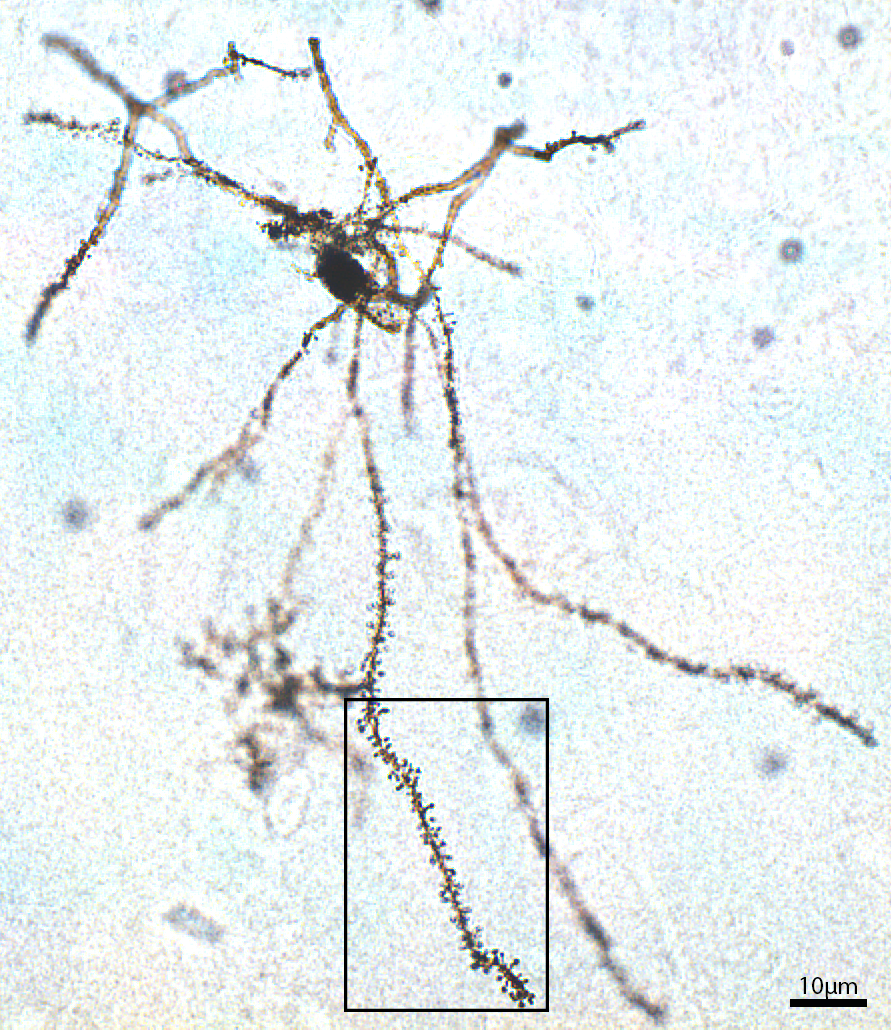

Supplement: Supplementary file 3 — Source data Fig. 1 [file 44321_2026_397_MOESM3_ESM.zip › Figure 1/Figure1E/Figure1E.TIF]

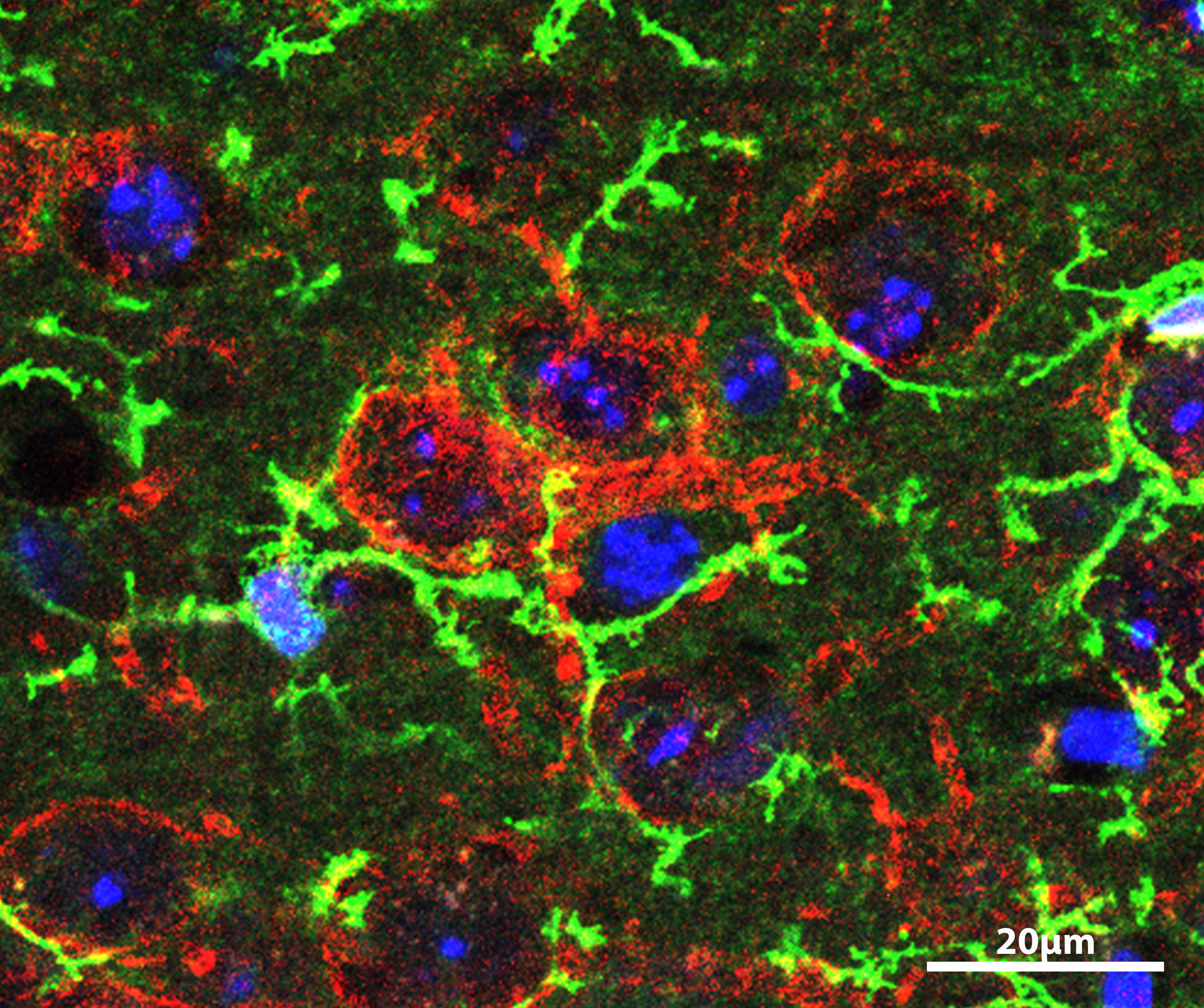

Supplement: Supplementary file 4 — Source data Fig. 2 [file 44321_2026_397_MOESM4_ESM.zip › Figure 2/Figure2H.tif]

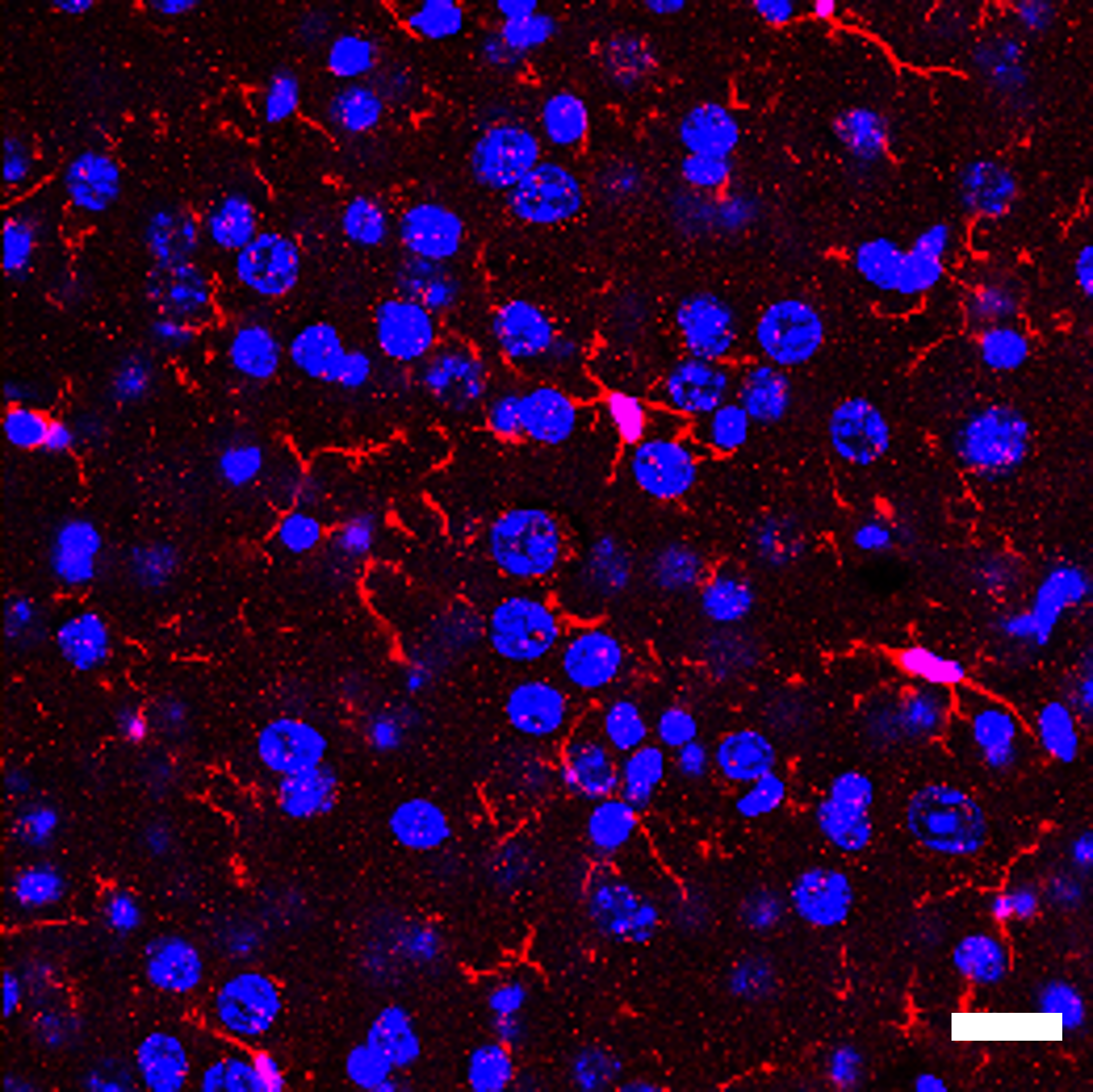

Supplement: Supplementary file 5 — Source data Fig. 3 [file 44321_2026_397_MOESM5_ESM.zip › Figure 3/Figure3D/Figure3D_left.tif]

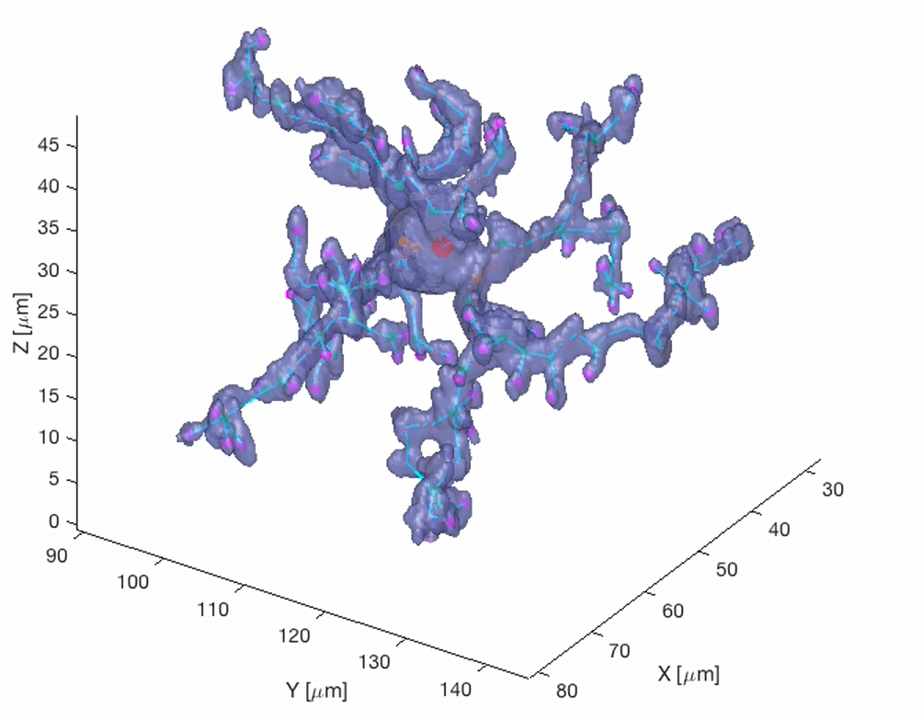

Supplement: Supplementary file 5 — Source data Fig. 3 [file 44321_2026_397_MOESM5_ESM.zip › Figure 3/Figure3D/Figure3D_right.tif]

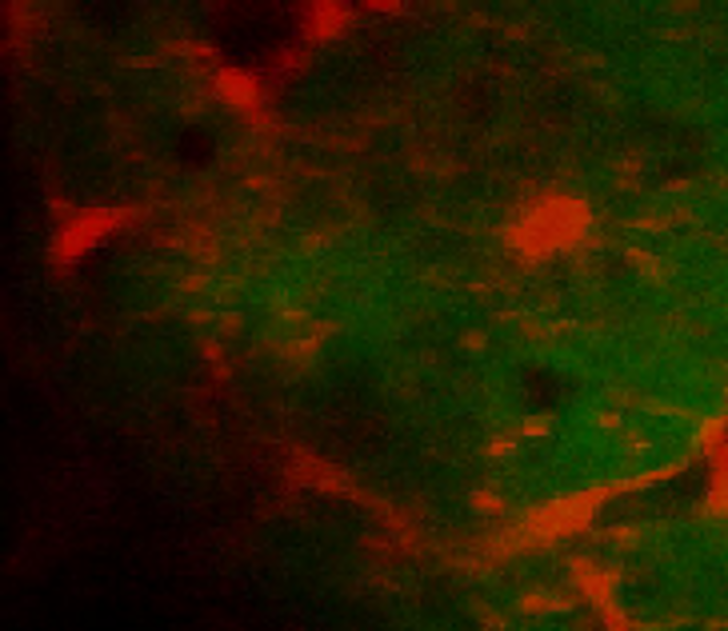

Supplement: Supplementary file 5 — Source data Fig. 3 [file 44321_2026_397_MOESM5_ESM.zip › Figure 3/Figure3C/Figure3C_3min.tif]

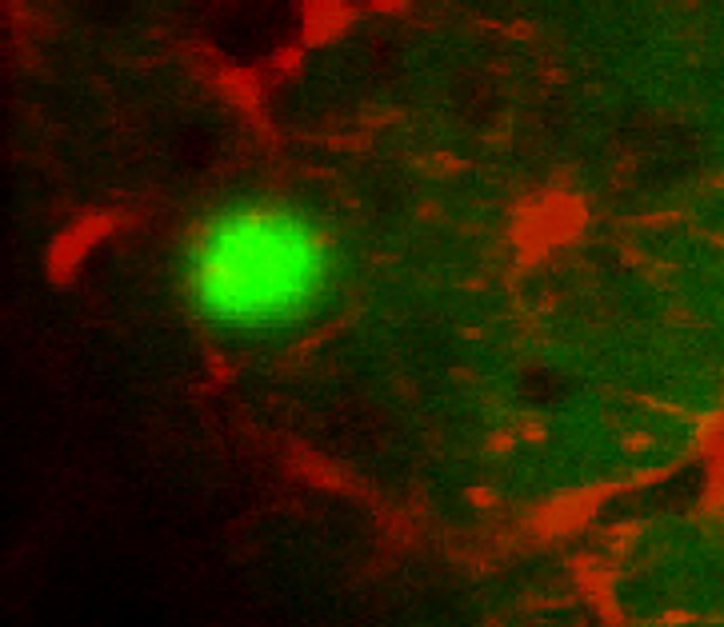

Supplement: Supplementary file 5 — Source data Fig. 3 [file 44321_2026_397_MOESM5_ESM.zip › Figure 3/Figure3C/Figure3C_1min.tif]

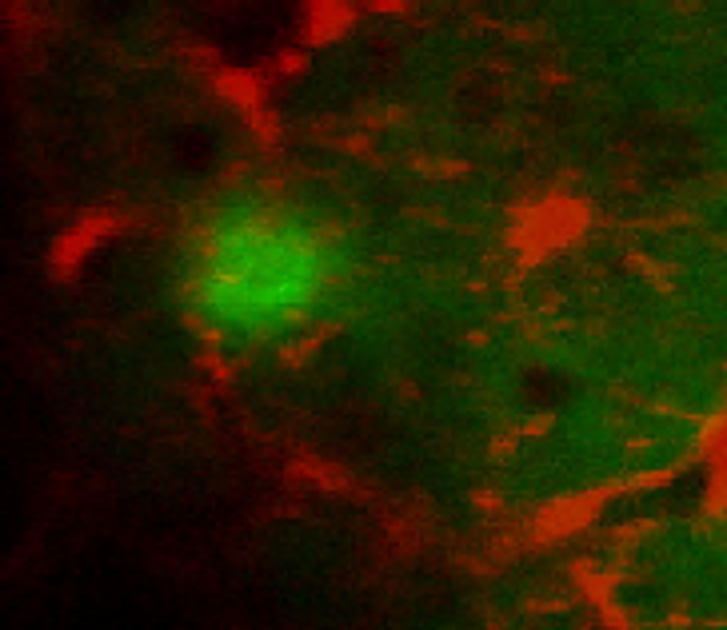

Supplement: Supplementary file 5 — Source data Fig. 3 [file 44321_2026_397_MOESM5_ESM.zip › Figure 3/Figure3C/Figure3C_2min.tif]

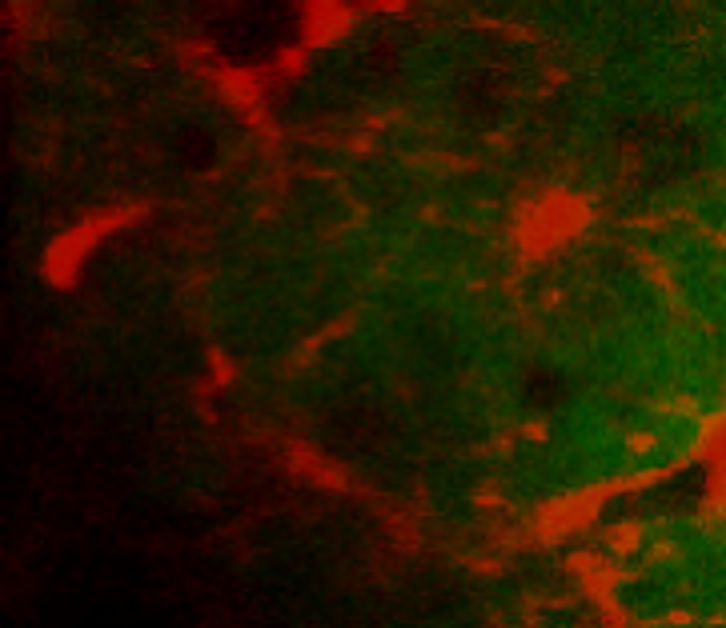

Supplement: Supplementary file 5 — Source data Fig. 3 [file 44321_2026_397_MOESM5_ESM.zip › Figure 3/Figure3C/Figure3C_0min.tif]

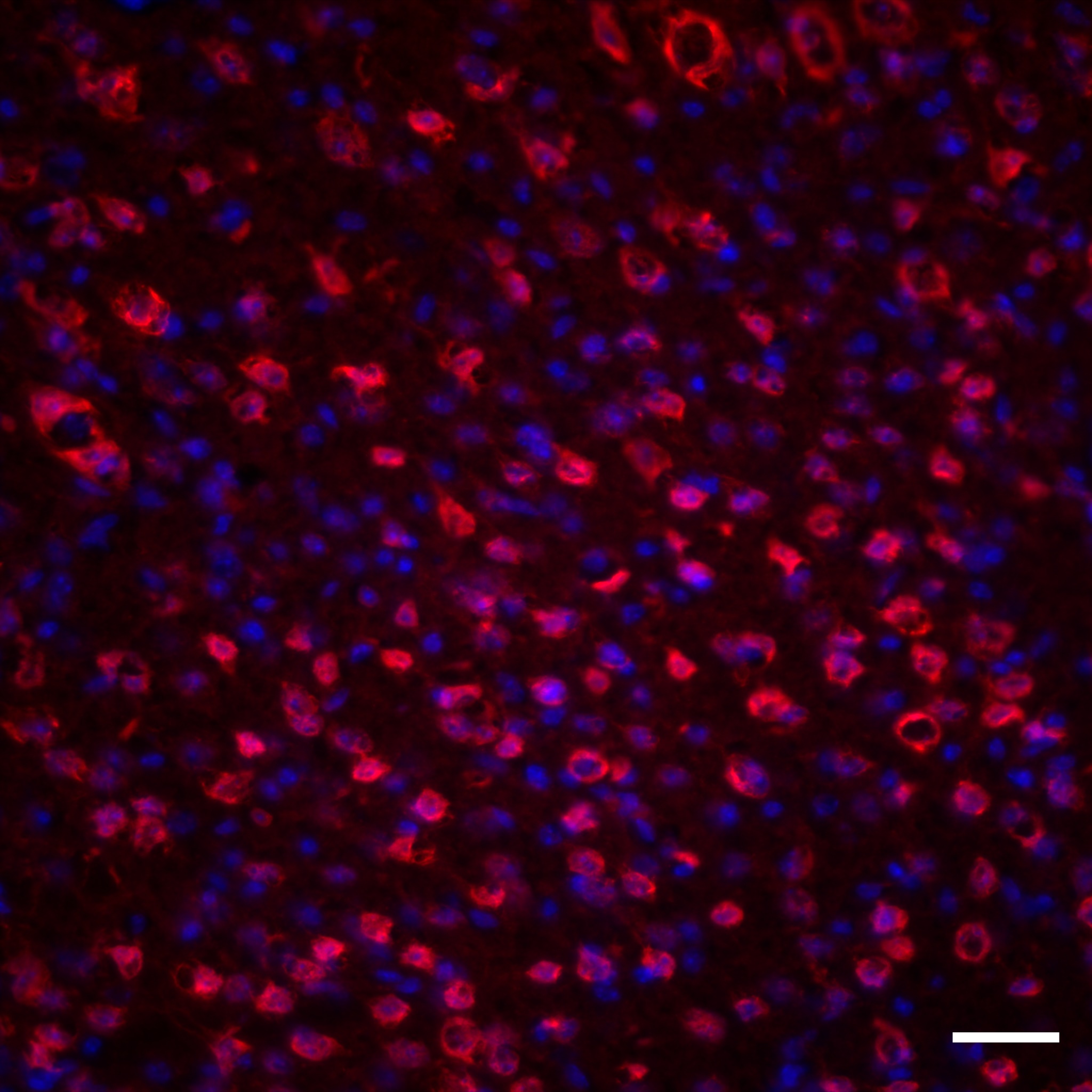

Supplement: Supplementary file 6 — Figure EV Source Data [file 44321_2026_397_MOESM6_ESM.zip › FIgure EV Source data/FigureEV1F/FigureEV1F.jpg]

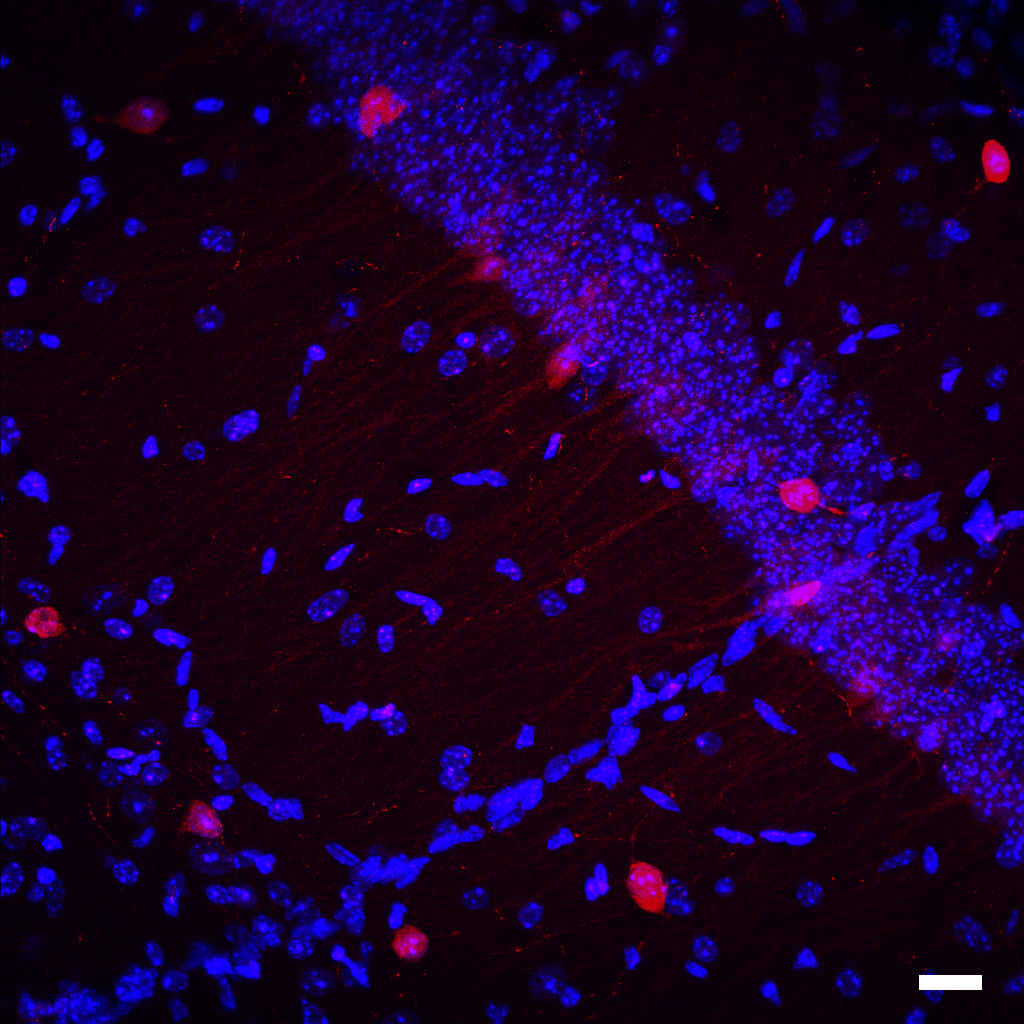

Supplement: Supplementary file 6 — Figure EV Source Data [file 44321_2026_397_MOESM6_ESM.zip › FIgure EV Source data/FigureEV1C/hypoxyprbe_TUNEL_AfterTIA_40x_zoom_ipsi_hipp_Maximum intensity projection.tif]

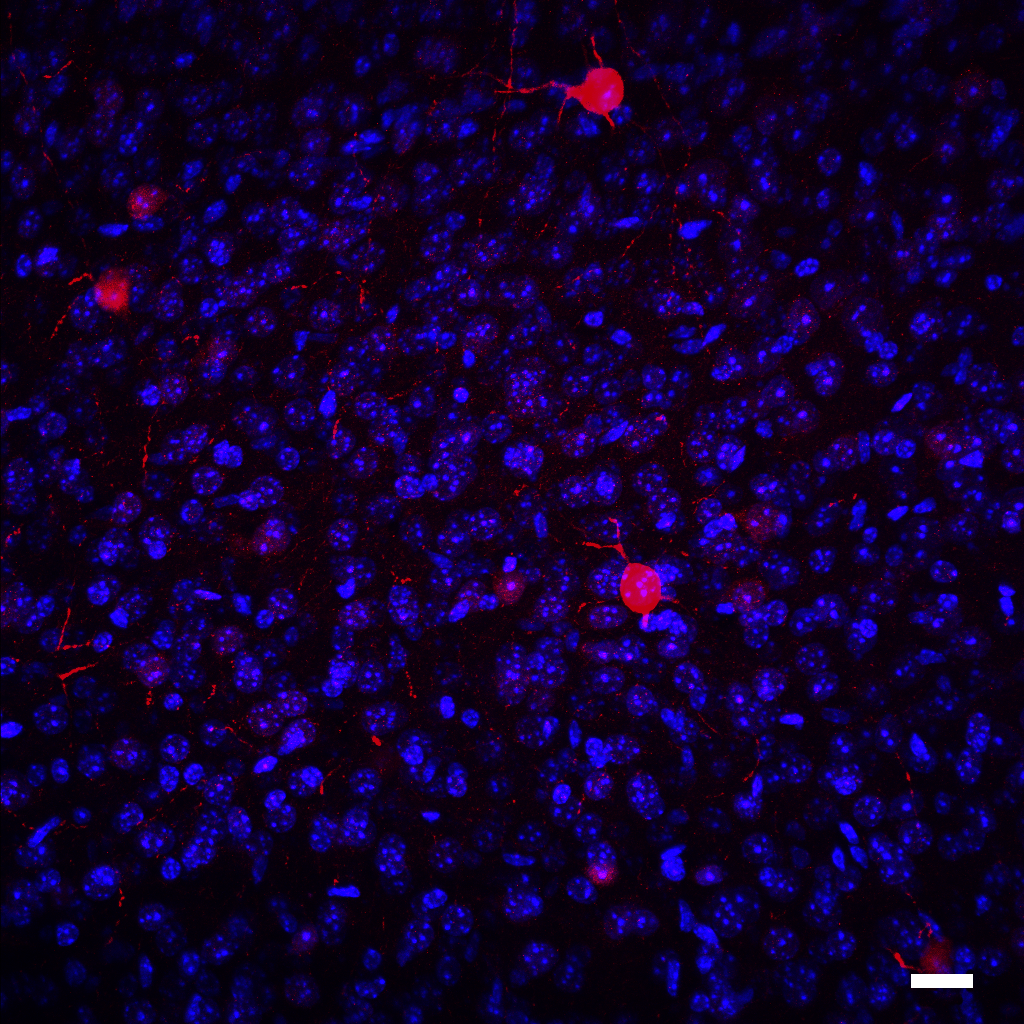

Supplement: Supplementary file 6 — Figure EV Source Data [file 44321_2026_397_MOESM6_ESM.zip › FIgure EV Source data/FigureEV1C/hypoxyprbe_TUNEL_AfterTIA_40x_zoom_ipsi_cx3_Maximum intensity projection.tif]

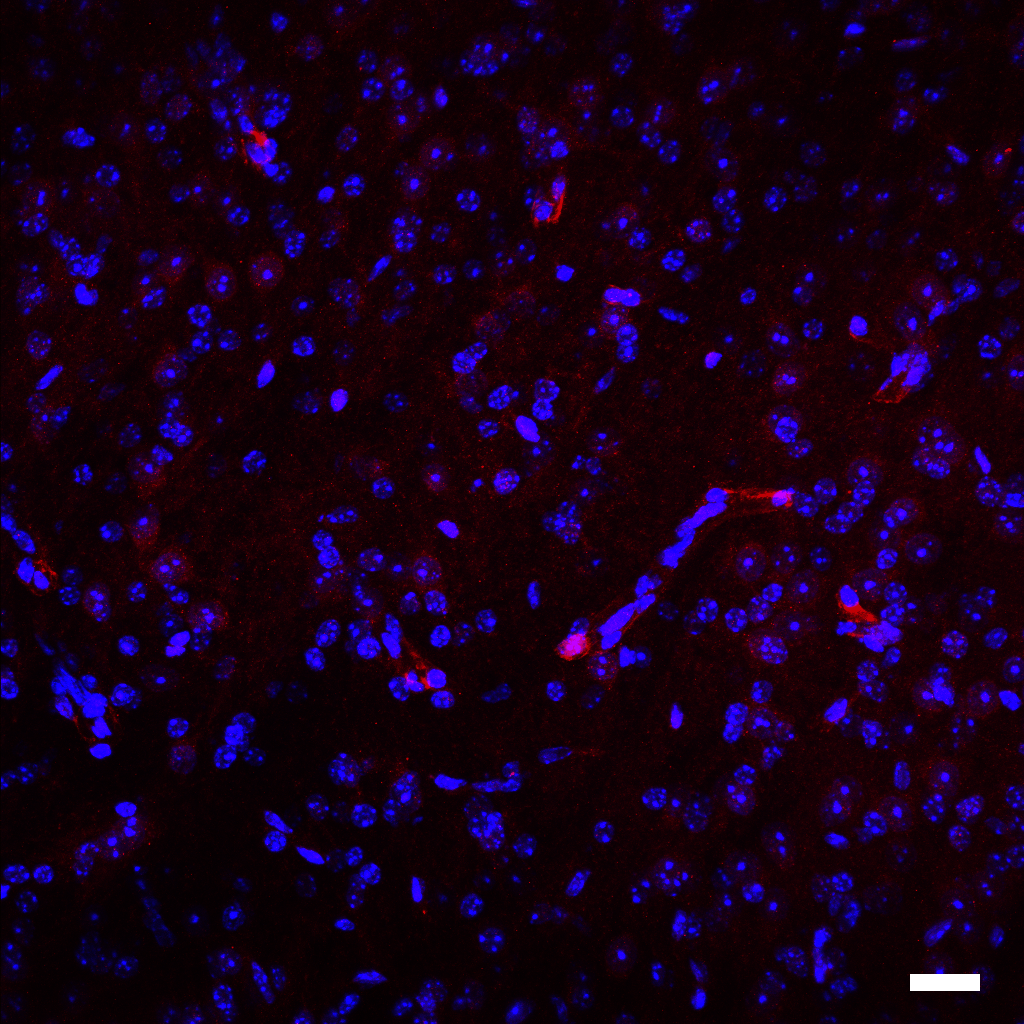

Supplement: Supplementary file 6 — Figure EV Source Data [file 44321_2026_397_MOESM6_ESM.zip › FIgure EV Source data/FigureEV1C/hypoxyprbe_TUNEL_AfterTIA_40x_zoom_ipsi_striatum_Maximum intensity projection.tif]

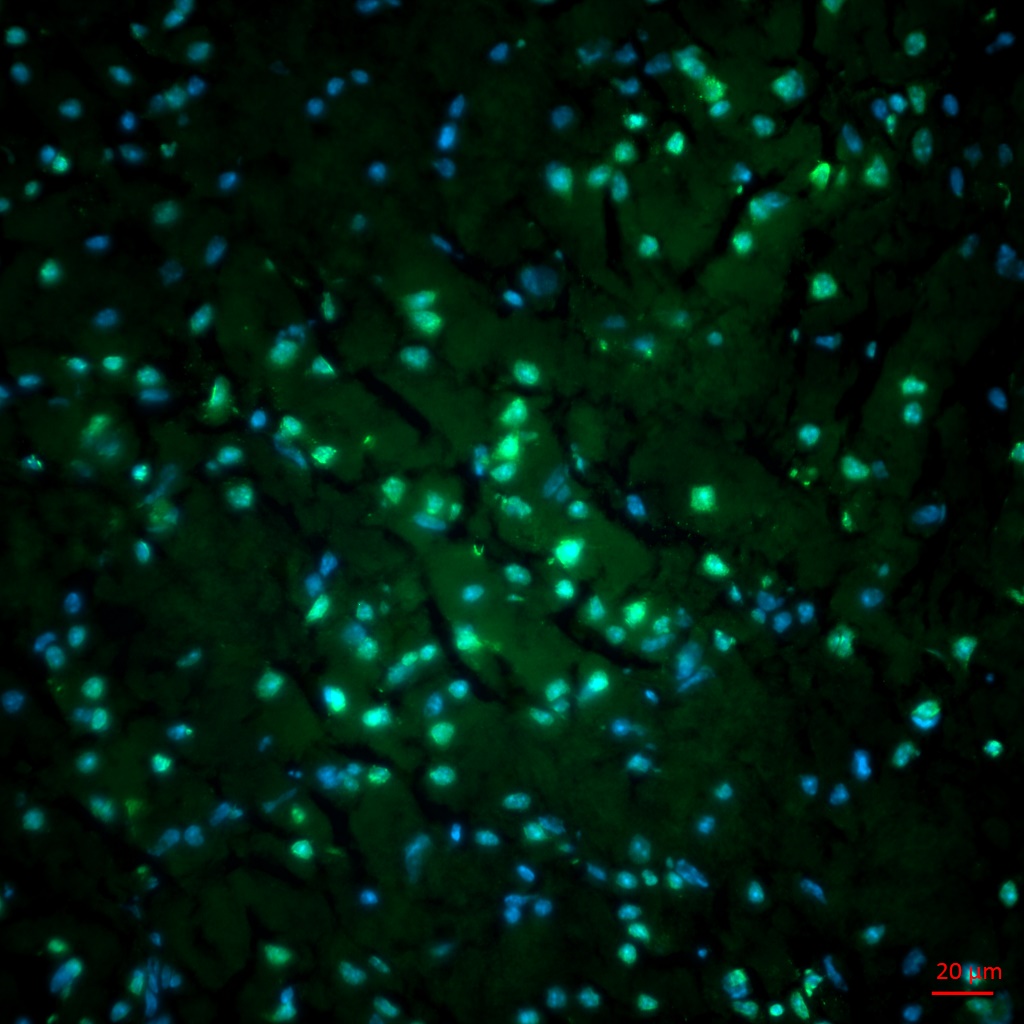

Supplement: Supplementary file 6 — Figure EV Source Data [file 44321_2026_397_MOESM6_ESM.zip › FIgure EV Source data/FigureEV1D/Tunel_30minTIA_40x_c1+2.jpg]

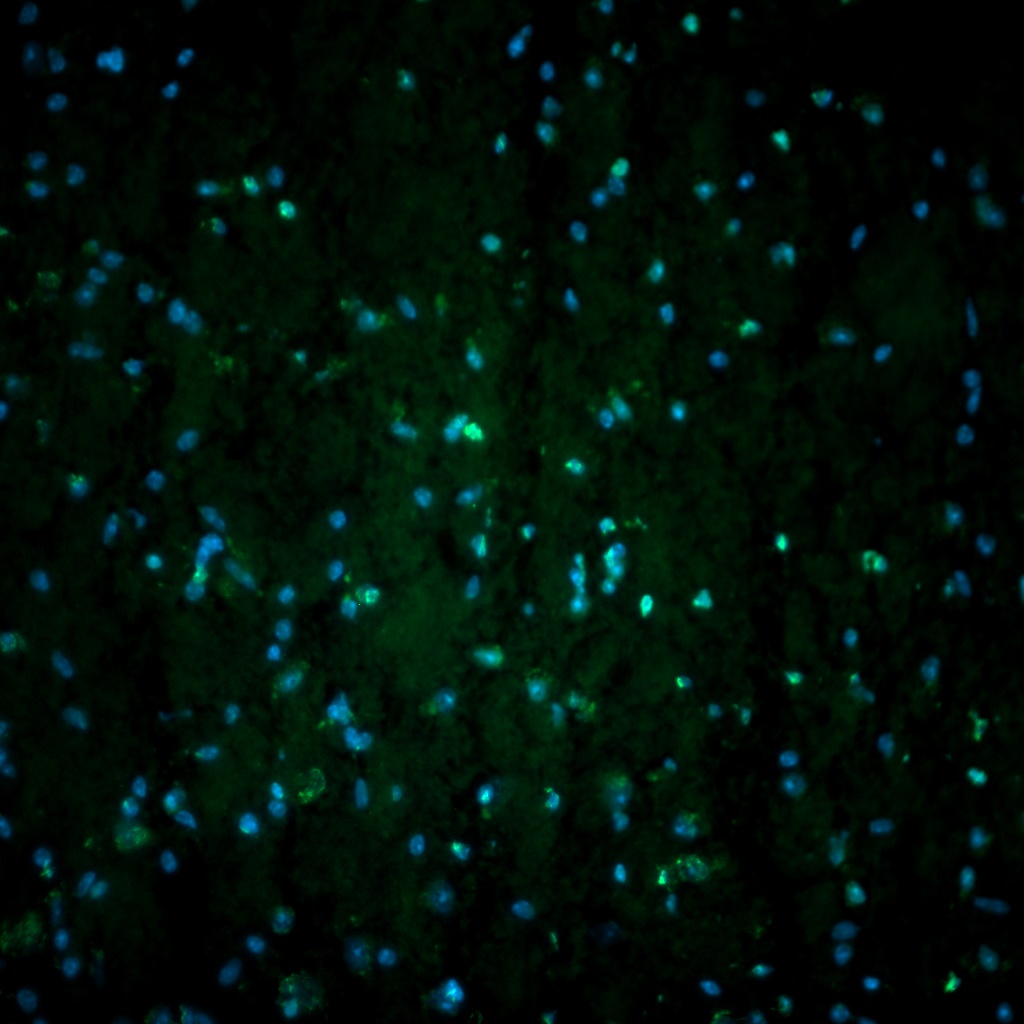

Supplement: Supplementary file 6 — Figure EV Source Data [file 44321_2026_397_MOESM6_ESM.zip › FIgure EV Source data/FigureEV1D/Tunel_15minTIA_40x_c_c1+2.jpg]

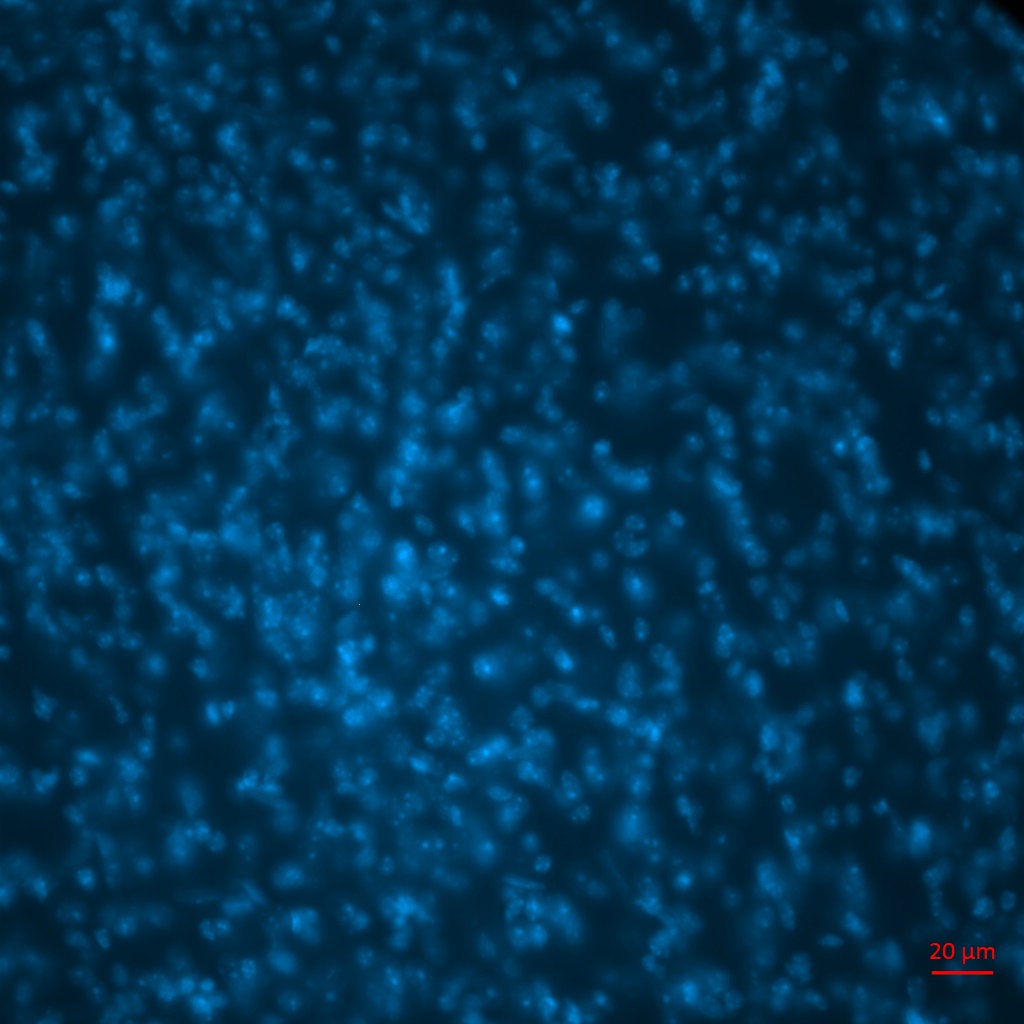

Supplement: Supplementary file 6 — Figure EV Source Data [file 44321_2026_397_MOESM6_ESM.zip › FIgure EV Source data/FigureEV1D/Tunel_ShamTIA_40x_c1+2.jpg]

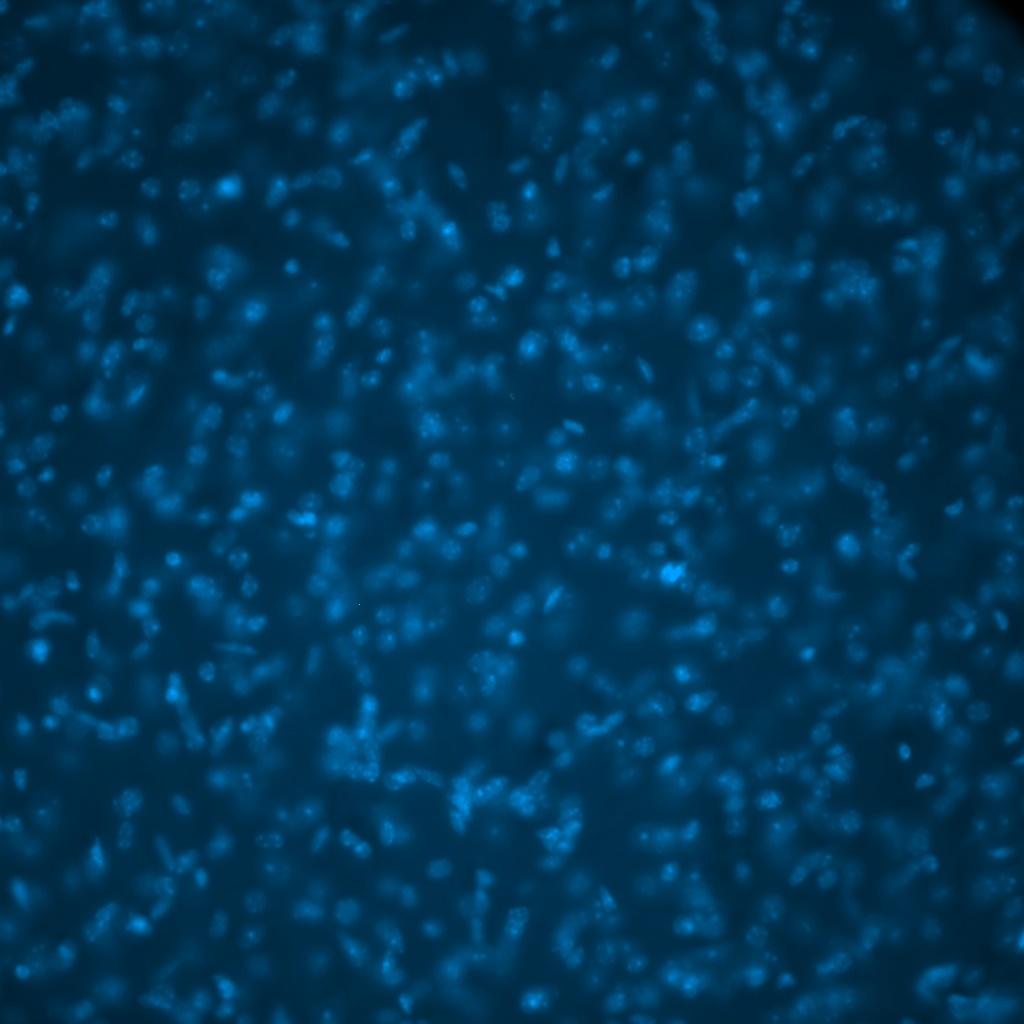

Supplement: Supplementary file 6 — Figure EV Source Data [file 44321_2026_397_MOESM6_ESM.zip › FIgure EV Source data/FigureEV1D/Tunel_5minTIA_40x_c1+2.jpg]

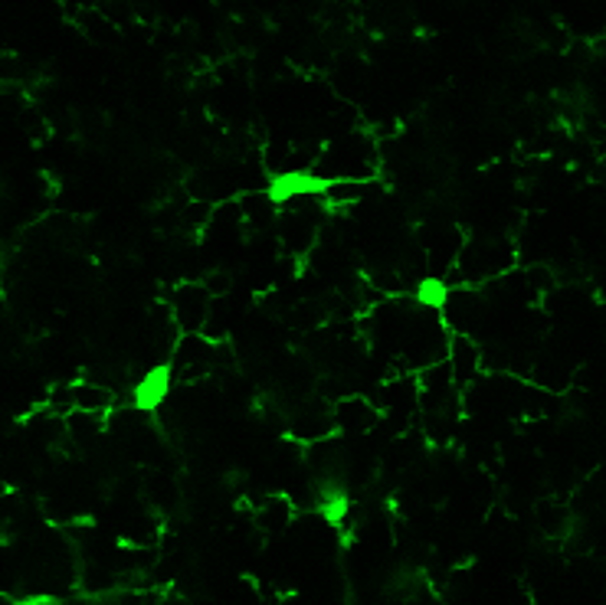

Supplement: Supplementary file 6 — Figure EV Source Data [file 44321_2026_397_MOESM6_ESM.zip › FIgure EV Source data/FigureEV5C/Figure EV5C_BL.tif]

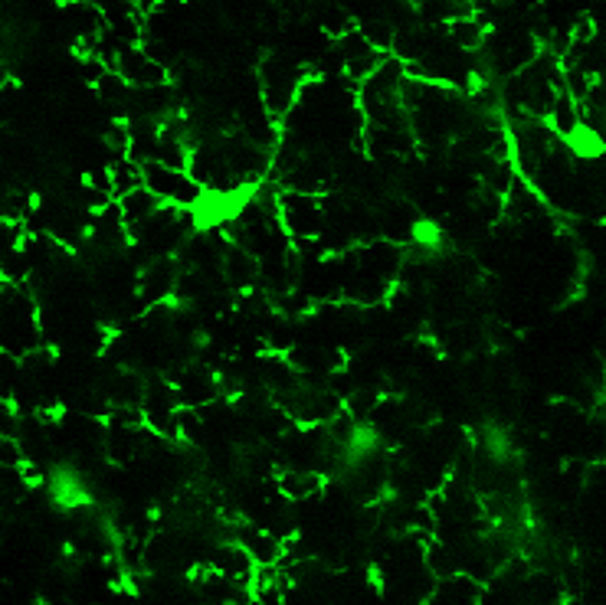

Supplement: Supplementary file 6 — Figure EV Source Data [file 44321_2026_397_MOESM6_ESM.zip › FIgure EV Source data/FigureEV5C/Figure EV5C_24h.tif]
