# Supplementary material for: Blocking microglial reactivity via purinergic receptors prevents subacute cognitive deficits after TIA
Source: EMBO Mol Med. 2026 Mar 20;18(4):1150–73. doi: 10.1038/s44321-026-00397-6 (PMC13083932; doi:10.1038/s44321-026-00397-6)
Supplement: Supplementary file 7 — Expanded View Figures [file 44321_2026_397_MOESM7_ESM.pdf]

# Expanded View Figures

**Figure EV1. Characterization of the TIA model.**

General and focal Neuroscore at different time points after TIA in (**A**) males ($n = 15$ per group; General Neuroscore: 4 h: $p$ value = 0.0004, 24 and 48 h:$p$ value <0.0001; Focal Neuroscore: 4, 24, and 48 h:$p$ value <0.0001, 3 d:$p$ value = 0.0012) and (**B**) females ($n = 5$ per group; General Neuroscore: 4, 24, 48 h and 3 d:$p$ value <0.0001; Focal Neuroscore: 4, 24 h: $p$ value <0.0001, 48 h:$p$ value = 0.0002, 3 d:$p$ value = 0.045). (**C**) Representative image for brain hypoxia (hypoxyprobe in red) and cell death ($TUNEL^+$ in green) 24 h after TIA in males in cortex (1), hippocampus (2) and striatum (3), scale bar = 20 µm. (**D**) Representative $TUNEL^+$ staining and quantification 24 h after different MCA occlusion times in males ($n = 5$ per group), scale bar = 20 µm. (**E**) Blood–brain barrier integrity was measured by extravasated Evans Blue per gram of the brain tissue 24 h after different ischemia times in males. Black = ipsilateral hemisphere, red = contralateral hemisphere ($n = 5$ per group; 15 and 30 min ischemia: $p$ value = 0.0079). (**F**) Representative image and analysis of neural cells (NeuN+) in different brain regions in males (DG dentate gyrus), scale bar = 20 µm. (**G**) Representative image and analysis of glucose metabolism using in vivo positron-emission tomography 24 h after TIA or Sham males ($n = 6$ per group). 1-Right striatum, 2-Left striatum, 3-Cortex, 4-Right hippocampus, 5-Left hippocampus, 6-Thalamus, 7-Carebelum, 8-Basal forebrain/septum, 9-Hypothalamus, 10-Right amygdala, 11-Left amygdala, 12-Brainstem, 13- Central gray, 14-Superior colliculi, 15-Olfactory bulb, 16-Midbrain right, 17-Midbrain left, 18- Inferior colliculi left, 19-Inferior colliculi right. (**H**) Representative scheme of the microperfusion system and protein intensity over time before (BL) and after TIA in males ($n = 5$ per group). Statistical tests: (**A**, **B**, **E**, **F**) two-way ANOVA, corrected for multiple comparisons using two-stage step-up method of Benjamin Kriegel. (**H**) Two-way ANOVA, Fisher's exact testing. Error bars indicate ±SD. *$p < 0.05$. Source data are available online for this figure.

© The Author(s)

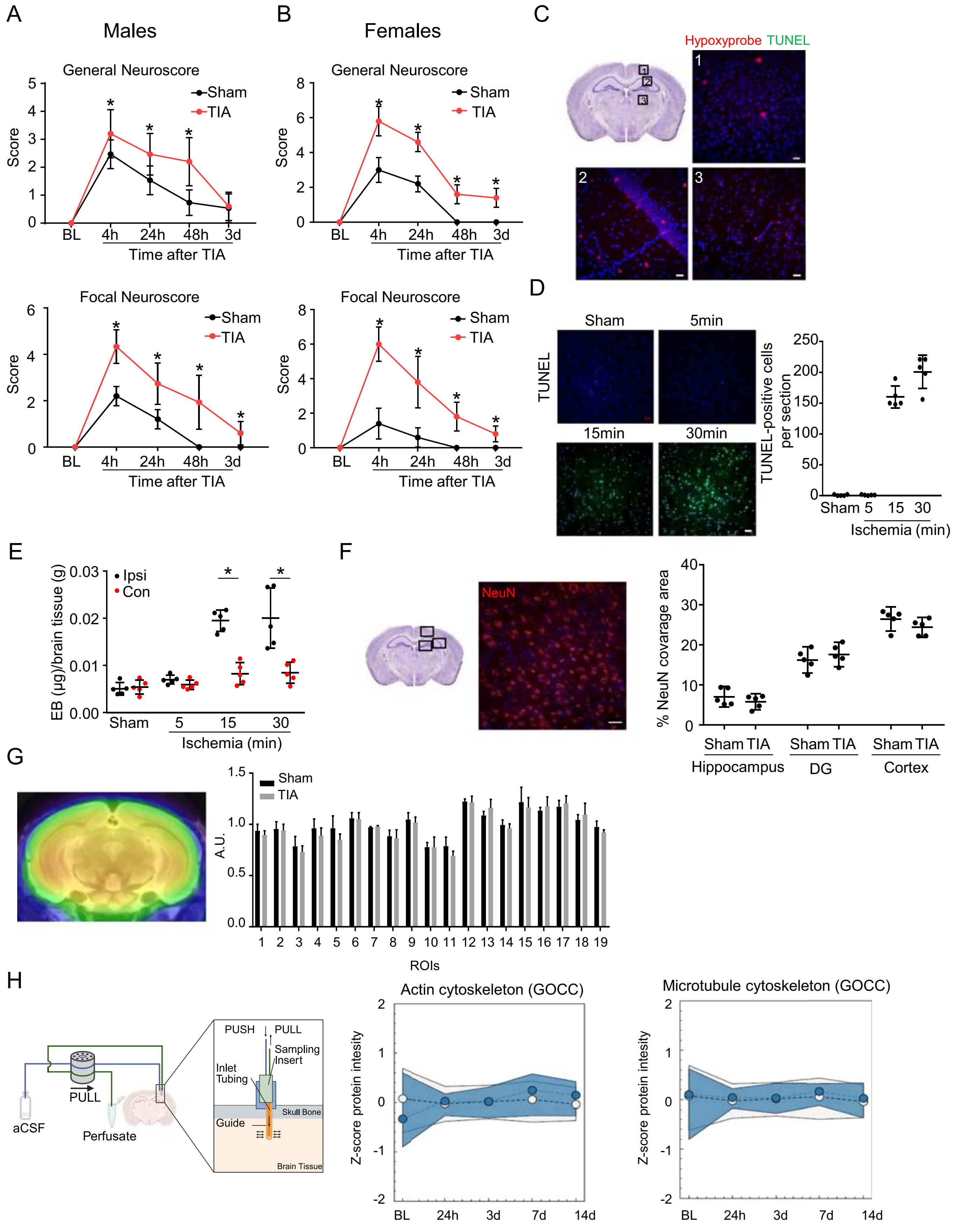
A
Males
General Neuroscore
Sham
TIA
Score
BL 4h 24h 48h 3d
Time after TIA
Focal Neuroscore
B
Females
C
Hypoxyprobe TUNEL
D
Sham 5min 15min 30min
TUNEL
TUNEL-positive cells per section
Sham 5 15 30
Ischemia (min)
E
EB (µg)/brain tissue (g)
Ipsi
Con
F
NeuN
% NeuN covarage area
Sham TIA
Hippocampus
DG
Cortex
G
A.U.
ROIs
H
PUSH PULL
Sampling Insert
Inlet Tubing
Skull Bone
Guide
Brain Tissue
PULL
aCSF
Perfusate
Actin cytoskeleton (GOCC)
Microtubule cytoskeleton (GOCC)
Z-score protein intesity
BL 24h 3d 7d 14d

 © The Author(s)

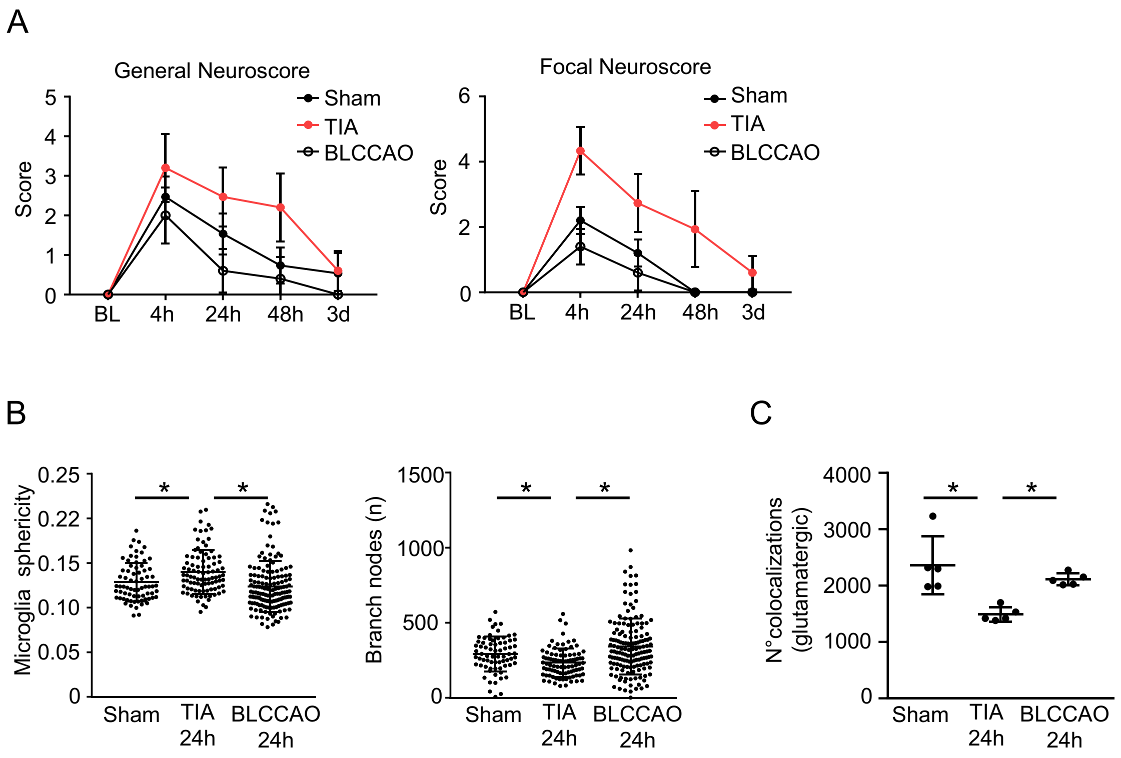


**Figure EV2. TIA vs BLCCAO.**

(**A**) General and focal Neuroscore at different time points after TIA, Sham and BLCCAO male animals ($n = 5$ per group). (**B**) Microglia morphology analysis (sphericity and branch nodes) at different time points 24 h after TIA, Sham and BLCCAO male animals ($n = 5$ per group; Microglia sphericity: TIA:$p$ value = 0.0123 and BLCCAO:$p$ value <0.0001), Branch nodes: TIA:$p$ value = 0.0022 and BLCCAO:$p$ value <0.0001. (**C**) Quantification of colocalized glutamatergic presynaptic and postsynaptic particles 24 h after TIA, Sham, and BLCCAO male animals ($n = 5$ per group; TIA:$p$ value = 0.0140 and BLCCAO:$p$ value = 0.0400). Statistical tests: (**A–C**) two-way ANOVA, corrected for multiple comparisons using two-stage step-up method of Benjamin Kriegel. Error bars indicate ±SD. $*p < 0.05$.

© The Author(s)

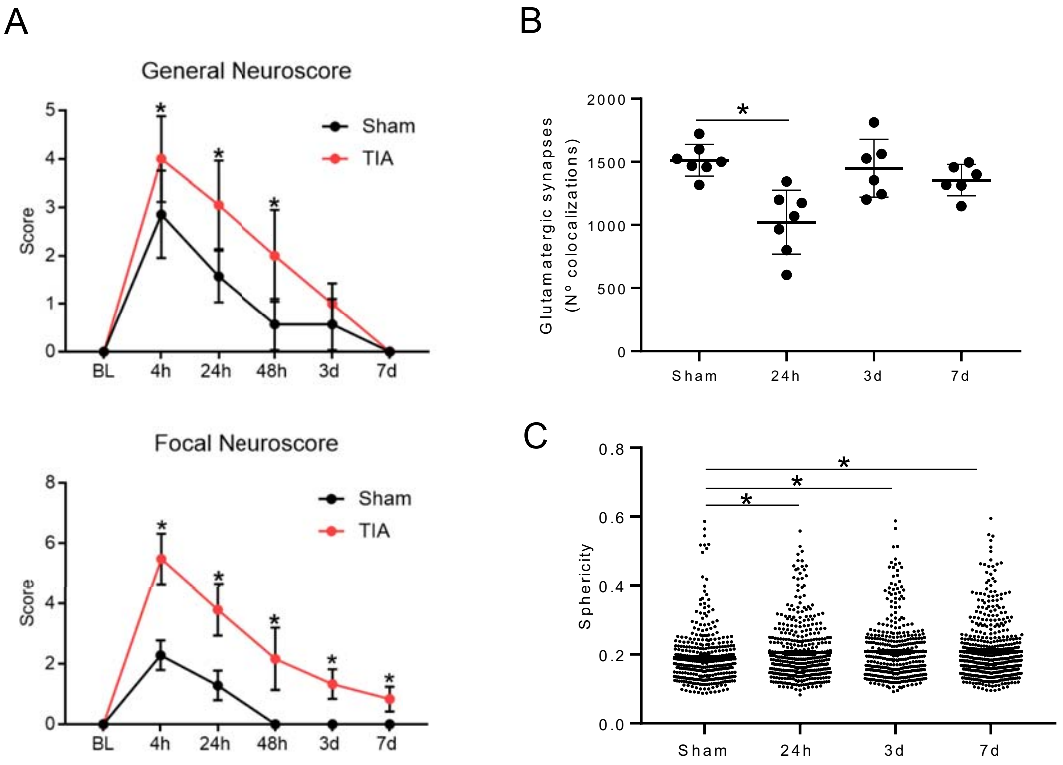


**Figure EV3. TIA in aging.**

(**A**) General and focal Neuroscore at different time points after TIA in male aging animals (20 months) (Sham: $n = 7$; TIA: BL-24 h $n = 19$, 48 h-3d $n = 12$, 7 d $n = 6$; General neuroscore: 4 h: $p$ value = 0.0002, 24 and 48 h:$p$ value <0.0001; Focal Neuroscore: 4, 24, and 48 h:$p$ value <0.0001, 3 d:$p$ value = 0.0034). (**B**) Quantification of colocalized glutamatergic presynaptic and postsynaptic particles at different time points after TIA in male aging animals (20 months) (Sham: $n = 7$; TIA: 24 h $n = 7$, 3d–7d $n = 6$; 24 h:$p$ value = 0.0003). (**C**) Microglia morphology analysis (sphericity) at different time points after TIA in male aging animals (20 months) (Sham: $n = 7$; TIA: 24 h $n = 7$, 3d–7d $n = 6$; 24 h:$p$ value = 0.0083, 3 d:$p$ value = 0.0027, 7 d:$p$ value = 0.0011). Statistical tests: (**A–C**) two-way ANOVA, corrected for multiple comparisons using two-stage step-up method of Benjamin Kriegel. Error bars indicate ±SD. *$p < 0.05$.

 © The Author(s)

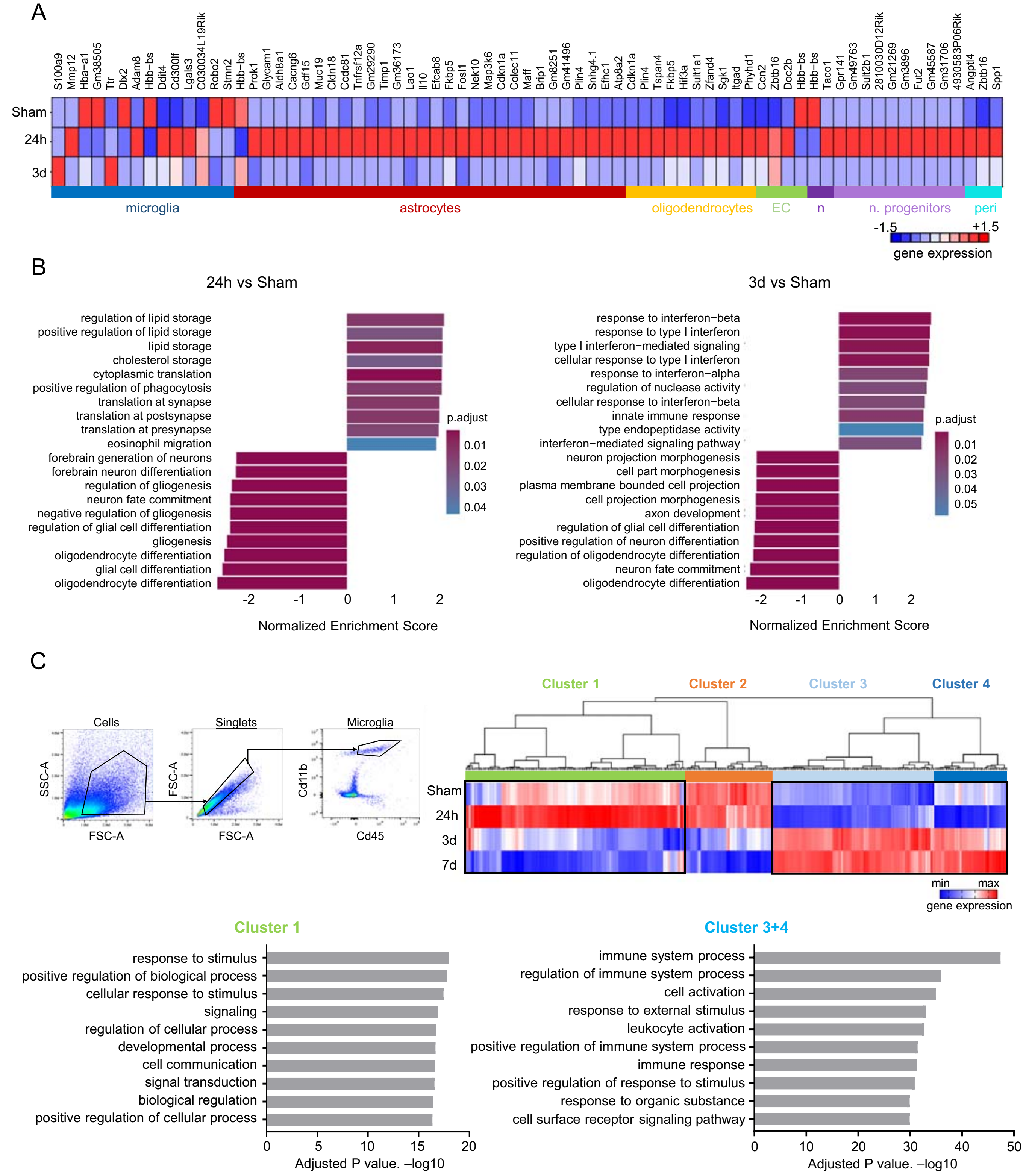
A
S100a9
Mmp12
Hba-a1
Gm38505
Ttr
Dlx2
Adam8
Hbb-bs
Ddit4
Cd300lf
Lgals3
C030034L19Rik
Robo2
Stmn2
Hbb-bs
Prok1
Glycam1
Aldh8a1
Cacng6
Gdf15
Muc19
Cldn18
Ccdc81
Tnfrsf12a
Gm29290
Timp1
Gm36173
Lao1
Il10
Efcab8
Fkbp5
Fosl1
Nek10
Map3k6
Cdkn1a
Colec11
Maff
Brip1
Gm8251
Gm41496
Plin4
Snhg4.1
Efhc1
Atp8a2
Cdkn1a
Plin4
Tspan4
Fkbp5
Hif3a
Sult1a1
Zfand4
Sgk1
Itgad
Phyhd1
Ccn2
Zbtb16
Doc2b
Hbb-bs
Hbb-bs
Taco1
Gpr141
Gm49763
Sult2b1
2810030D12Rik
Gm21269
Gm3896
Fut2
Gm45587
Gm31706
4930583P06Rik
Angptl4
Zbtb16
Spp1
Sham
24h
3d
microglia
astrocytes
oligodendrocytes
EC
n
n. progenitors
peri
-1.5
+1.5
gene expression
B
24h vs Sham
regulation of lipid storage
positive regulation of lipid storage
lipid storage
cholesterol storage
cytoplasmic translation
positive regulation of phagocytosis
translation at synapse
translation at postsynapse
translation at presynapse
eosinophil migration
forebrain generation of neurons
forebrain neuron differentiation
regulation of gliogenesis
neuron fate commitment
negative regulation of gliogenesis
regulation of glial cell differentiation
gliogenesis
oligodendrocyte differentiation
glial cell differentiation
oligodendrocyte differentiation
p.adjust
0.01
0.02
0.03
0.04
-2
-1
0
1
2
Normalized Enrichment Score
3d vs Sham
response to interferon−beta
response to type I interferon
type I interferon−mediated signaling
cellular response to type I interferon
response to interferon−alpha
regulation of nuclease activity
cellular response to interferon−beta
innate immune response
type endopeptidase activity
interferon−mediated signaling pathway
neuron projection morphogenesis
cell part morphogenesis
plasma membrane bounded cell projection
cell projection morphogenesis
axon development
regulation of glial cell differentiation
positive regulation of neuron differentiation
regulation of oligodendrocyte differentiation
neuron fate commitment
oligodendrocyte differentiation
p.adjust
0.01
0.02
0.03
0.04
0.05
-2
-1
0
1
2
Normalized Enrichment Score
C
Cells
SSC-A
FSC-A
Singlets
FSC-A
FSC-A
Microglia
Cd11b
Cd45
Cluster 1
Cluster 2
Cluster 3
Cluster 4
Sham
24h
3d
7d
min
max
gene expression
Cluster 1
response to stimulus
positive regulation of biological process
cellular response to stimulus
signaling
regulation of cellular process
developmental process
cell communication
signal transduction
biological regulation
positive regulation of cellular process
0
5
10
15
20
Adjusted P value. –log10
Cluster 3+4
immune system process
regulation of immune system process
cell activation
response to external stimulus
leukocyte activation
positive regulation of immune system process
immune response
positive regulation of response to stimulus
response to organic substance
cell surface receptor signaling pathway
0
10
20
30
40
50
Adjusted P value. –log10

**Figure EV4. The impact of TIA on microglia transcriptome.**

(**A**) Heat map of normalized and scaled (*z*-score) gene expression of significantly differentially up- and down-regulated genes in each cell type at 24 h and 3 d after TIA compared to Sham in males ($n = 3$ per group). (**B**) Gene set enrichment analyses (Biological Processes) of differentially regulated genes in microglial cells at 24 h and 3 d compared to Sham in males ($n = 3$ per group). (**C**) Representative FACS plot sort strategy for microglia-like cells isolation, cluster heat map showing ANOVA+ genes from Nanostring analysis from isolated microglia-like cells at different time points after TIA and pathway analysis from selected cluster 1 ($n = 3$ per group) and cluster 3 + 4 ($n = 3$ per group) in males. Statistical tests: (**A**) Wilcoxon rank sum test + bonferroni correction. (**B**) Genes ranked by log2 fold change, permutation-based GSEA, Benjamini–Hochberg correction, (**C**) Data were analyzed by ROSALIND® (https://rosalind.bio/). Significantly regulated genes were identified by ANOVA multiple sample testing (S0 = 0.1, permutation-based FDR = 0.05)

 © The Author(s)

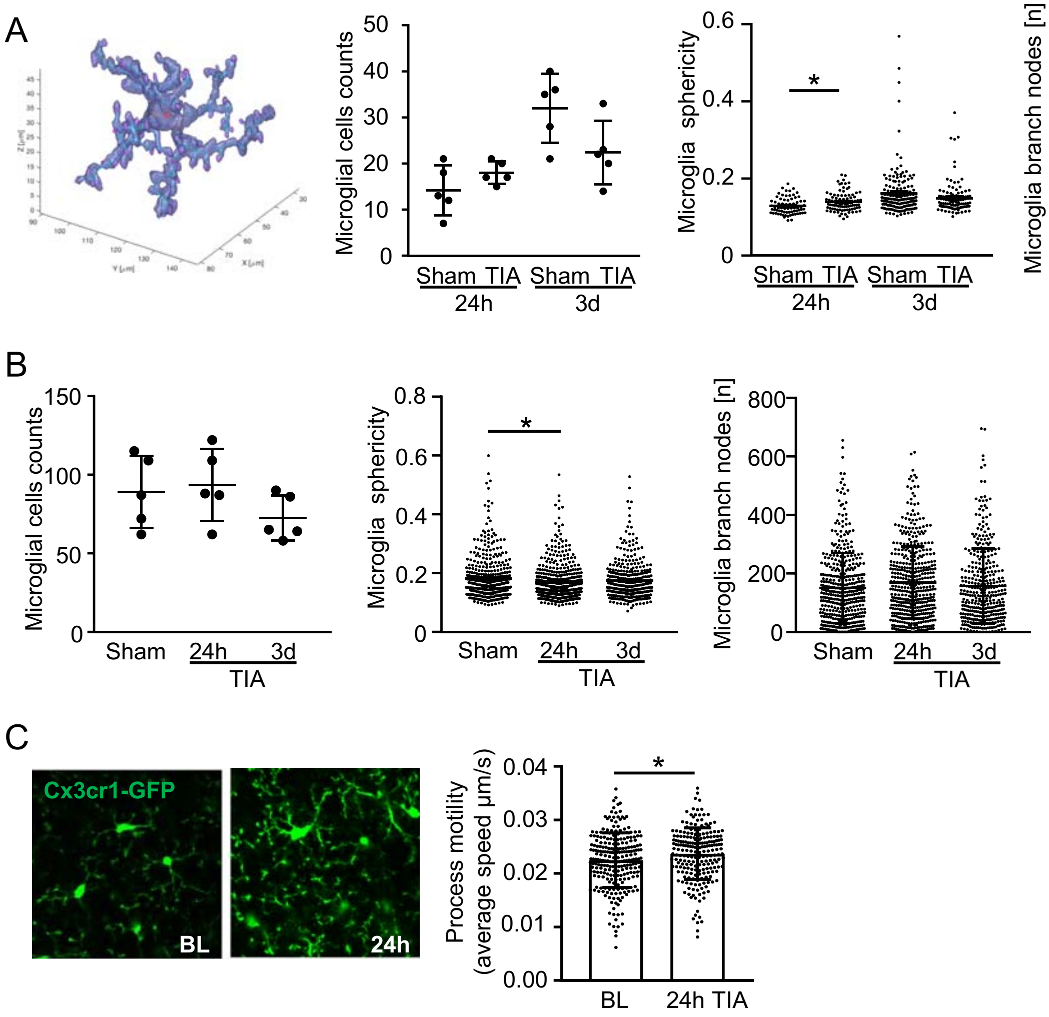


**Figure EV5. The impact of TIA on microglia morphology.**

(**A**) Representative image of 3D reconstructed microglia for microglia cells counts and microglia morphology analysis for two representative features: sphericity and branches nodes at 24 h after TIA ($n = 5$ per group; sphericity 24 h:*p* value = 0.019; branch nodes 24 h:*p* value = 0.022) in males and (**B**) females ($n = 5$ per group; sphericity 24 h:*p* value = 0.0032). (**C**) Representative images of in vivo two-photon imaging of the microglia process motility and quantification at baseline (BL) and 24 h after TIA in males ($n = 8$ per group; 24 h:*p* value = 0.025). Statistical tests: (**A**, **C**) two-way ANOVA, corrected for multiple comparisons using two-stage step-up method of Benjamin Kriegel. (**B**) two-way Student's *t*-test. Error bars are mean ± SD. $^{*}P < 0.05$. Source data are available online for this figure.

© The Author(s)

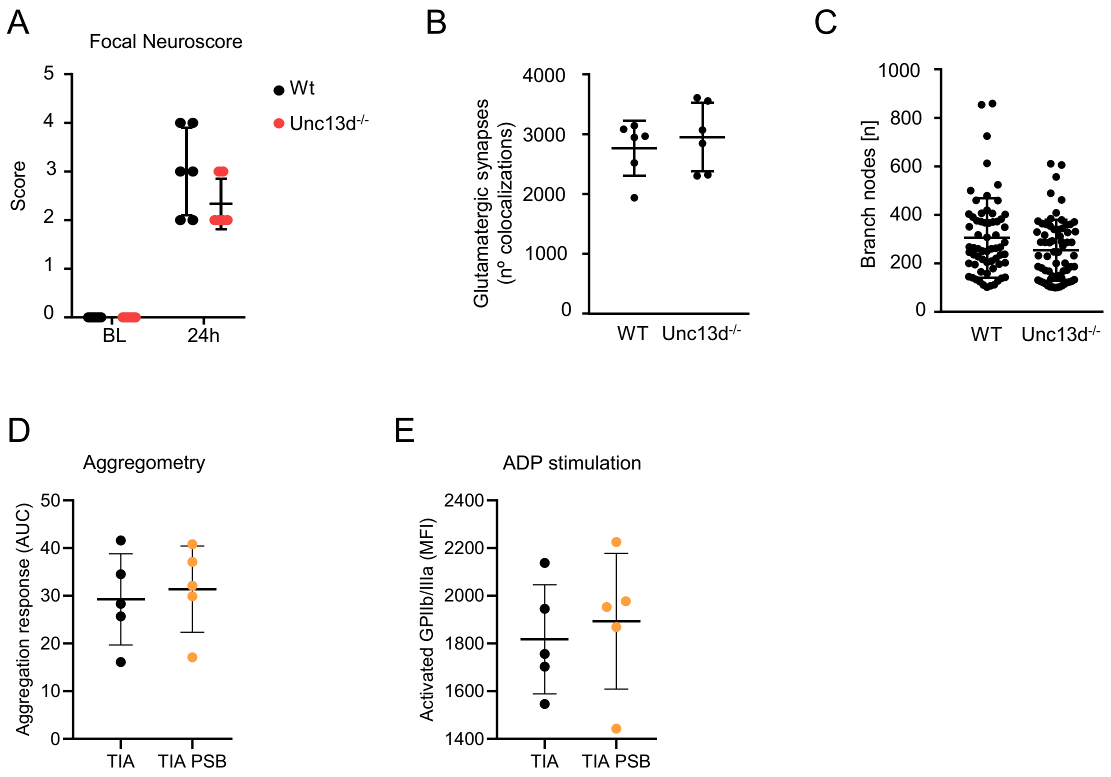


**Figure EV6. Platelets are not critical after a TIA.**

(**A**) Focal neuroscore, (**B**) glutamatergic synapses, and (**C**) microglia branch nodes analysis of Unc13KO and littermates wild-type (WT) animals 24 h after TIA in males. (**D**) Platelet aggregation and (**E**) activation in TIA in vehicle (Veh) or P2Y12Ri (-inhibitor) treated male animals ($n = 5$ per group). Statistical tests: (**A–E**) two-way Student's *t*-test. Error bars are mean ± SD.

 © The Author(s)
